# Flexible Monitoring, Diagnosis, and Therapy by Microneedles with Versatile Materials and Devices toward Multifunction Scope

**DOI:** 10.34133/research.0128

**Published:** 2023-04-28

**Authors:** Shuo Wang, Mengmeng Zhao, Yibo Yan, Peng Li, Wei Huang

**Affiliations:** Frontiers Science Center for Flexible Electronics (FSCFE), Xi’an Institute of Flexible Electronics (IFE) and Xi’an Institute of Biomedical Materials & Engineering, Northwestern Polytechnical University, Xi'an, China.

## Abstract

Microneedles (MNs) have drawn rising attention owing to their merits of convenience, noninvasiveness, flexible applicability, painless microchannels with boosted metabolism, and precisely tailored multifunction control. MNs can be modified to serve as novel transdermal drug delivery, which conventionally confront with the penetration barrier caused by skin stratum corneum. The micrometer-sized needles create channels through stratum corneum, enabling efficient drug delivery to the dermis for gratifying efficacy. Then, incorporating photosensitizer or photothermal agents into MNs can conduct photodynamic or photothermal therapy, respectively. Besides, health monitoring and medical detection by MN sensors can extract information from skin interstitial fluid and other biochemical/electronic signals. Here, this review discloses a novel monitoring, diagnostic, and therapeutic pattern by MNs, with elaborate discussion about the classified formation of MNs together with various applications and inherent mechanism. Hereby, multifunction development and outlook from biomedical/nanotechnology/photoelectric/devices/informatics to multidisciplinary applications are provided. Programmable intelligent MNs enable logic encoding of diverse monitoring and treatment pathways to extract signals, optimize the therapy efficacy, real-time monitoring, remote control, and drug screening, and take instant treatment.

## Introduction

Lethal contagions and massive other diseases are threatening human health and quality of living. Pharmaceuticals have to pass through the stratum corneum (SC) and viable epidermis before entering the capillaries and circulatory system [1]. Three routes are widely accepted for the passive diffusion of drugs, namely, through hair follicles (HFs), sweat ducts, and SC [2]. The SC is mainly formed by dead keratinocytes and prevents water loss from internal tissues and inhibits penetration of external substances, resulting in limited efficacy of conventional patches [3]. Besides, the other 2 routes are so inefficient that their contributions are almost negligible [4]. Thereby, pharmaceuticals tend to select low molecular weight (<500 Da) drugs to ensure high diffusion coefficients and appropriate lipophilicity, and only 10 to 20% of the drug can diffuse across the SC for successful transdermal delivery [5]. Hence, the advanced delivery devices are critically demanded for high-throughput transdermal drug delivery (TDD) and therapy across the barrier of SC.

Microneedles (MNs) generally consist of arrays of micrometer-sized needles with a height that ranged from 25 to 1,500 μm [6,7]. The microfabrication techniques of MNs employ valid materials and geometries that allow controlled drug delivery to the targeted subcutaneous layer beneath the SC [8]. The MNs can penetrate the SC and epidermis to create microchannels for drug delivery into dermis with minimally invasive and accurate control [9]. MNs are long enough to overcome the SC barrier, yet short enough not to irritate nerve endings, which are related to pain [10]. They are painless, precisely controlled, flexible, and easy to apply, with multiple advantages superior to traditional invasive injections and/or oral-based strategies [11]. In addition, other treatment approaches joining MNs are employed to achieve multifunctional therapy or treatment of diseases [12], including cancer [13], wound healing [14], diabetes [15], obesity [16], alopecia [17], transcutaneous immune system [18], etc. [19–21]. Incorporating photosensitizer or photothermal agents into MNs can conduct photodynamic or photothermal therapy (PTT), respectively. Specifically, photothermal agents can convert the adsorbed light into heat under near-infrared (NIR) laser irradiation that kills bacteria or targeted cells, while photodynamic therapy (PDT) yields reactive oxygen species (ROS) that can decompose target cells (e.g., microbial, bacteria, tumor, and pathogen) [22].

**Fig. 1.**
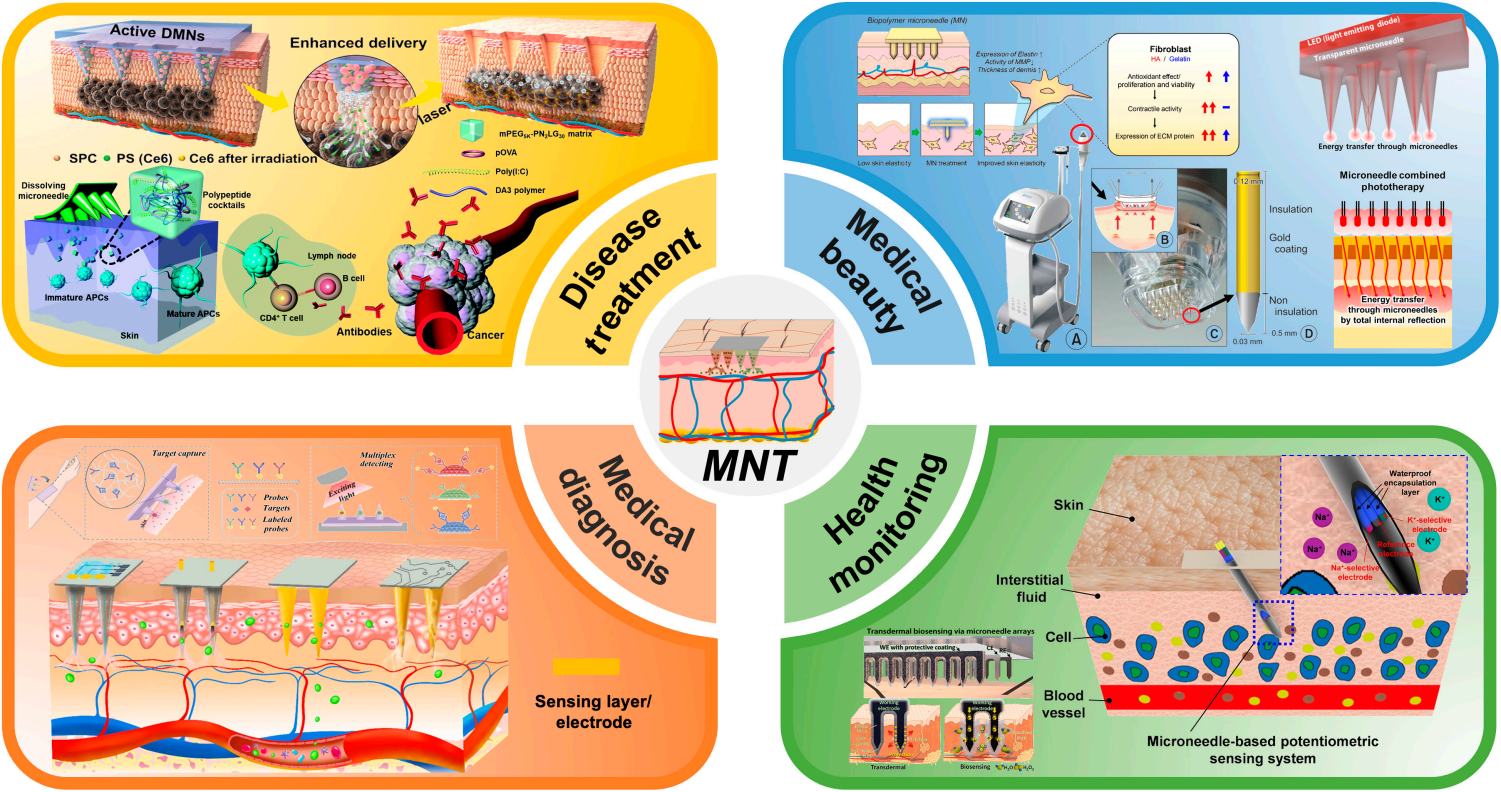
Schematic illustrations of MNT for disease treatment, medical diagnosis, health monitoring, and medical beauty. Reprinted with permission from [191, 240]. Copyright 2019, 2022 John Wiley & Sons. Reprinted with permission from [241]. Copyright 2020 The Royal Society of Chemistry. Reprinted with permission from [242, 243]. Copyright 2020, 2022 Elsevier. Reprinted with permission from [244, 245]. Copyright 2019, 2021 American Chemical Society. Reprinted with permission from [246]. Copyright 2019 Springer Nature. Reprinted with permission from [247]. Copyright 2021 Korean Society of Korean Cosmetic Surgery.

Microneedle therapy (MNT) refers to a diagnostic and therapeutic strategy based on material science and biomedical and flexible electronics/optoelectronics using MN platform for diagnosis and treatment of targeted diseases (e.g., wound healing, diabetes, obesity, alopecia, and tumor treating) via the multifunctionalization of MNs, involving medical diagnosis, health monitoring, medical beauty, transcutaneous immunization (TCI), and other multidisciplinary functions (Fig. 1). This review briefly unravels the classification and preparation of MNs, shedding light on the diverse applications of MNT in recent years and further prospects for multifunctional applications of real-time monitoring, remote control, diagnosis, drug screening, and therapy. Multifunctional MNs integrate multidisciplinary principles of biomedical engineering, material science, nanotechnology, electronic/optoelectronic devices, informatics, optogenetics, big data, artificial intelligence, artificial neural networks, human-machine interface, brain-computer interfaces, and logic encoding of diverse monitoring and treatment pathways to process different conditions and optimize therapy efficacy.

## Classification and Preparation of MNs

**Fig. 2.**
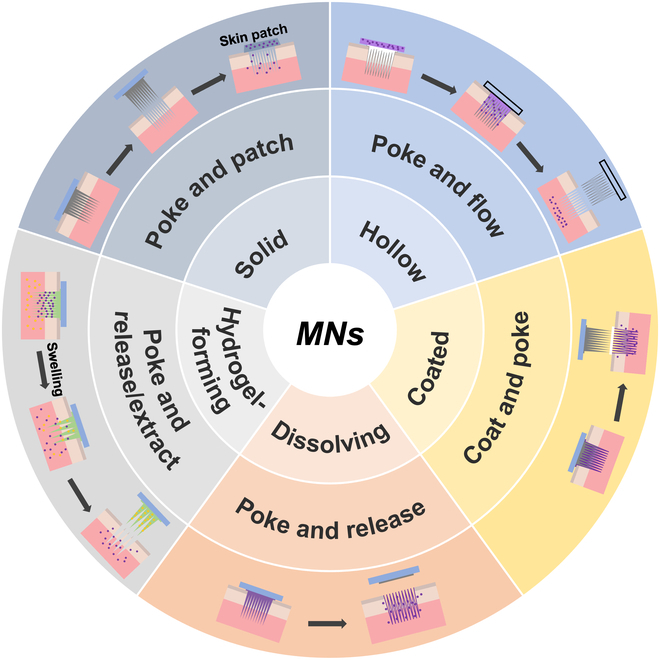
Classification and working modes of MNs. Solid MN acts on the skin and is then removed, forming microchannels in the SC, after which conventional drug formulations (patch, solution, latex, or gel) are applied to the skin, and the drug enters the subcutaneous layer from the microchannels by passive diffusion. After the coated MN is inserted into the skin, the drug in the coating will be dissolved and deposited in the skin. Hollow MNs deliver specific drugs into the skin by injecting a fluid formulation through a hollow needle inserted into the skin. The dissolving MNs dissolve and release the drug after contact with the skin ISF. HFMNs induce drug diffusion from the needle tip by absorbing ISF from the tissue.

The MN fabrication methods include lithography, cast micromolding, injection molding, hot embossing, droplet-born air blowing (DAB), drawing lithography, centrifugal lithography (CL), 3-dimensional (3D) printing methods, and so on. MNs are generally classified into solid MNs, coated MNs, hollow MNs, dissolving MNs, and hydrogel-forming MNs (HFMNs) (Fig. 2). Abundant studies have been reported on the incorporated functional materials and classification and preparation of MNs [23–26], and here, the comprehensive and concise refining of MN manufacture methods is exhibited as follows.

### Classification of MNs

#### Solid MNs

Solid MNs, the earliest type of MNs for TDD, are usually made up of silicon, metals, and polymers [27]. Practical applicability of solid MNs involves facile attachment to skin before taking off. The microchannels are created through the SC, and conventional drug formulations (patches, solutions, or gels) are then applied to the skin. The drugs diffuse through microchannels to target subcutaneous layer [28]. Through simultaneous etching of the front and back sides of the (100) silicon wafer in KOH solution, MNs with different aspect ratios could be prepared by using the (110) and (111) 54.7° angle planes, and hollow MNs could also be prepared by changing the mask [29]. Such process is inexpensive and scalable for manufacture in biomedical industry. Solid MNs have good mechanical properties, but once the needle is broken or damaged during the TDD process, the needle body will be trapped inside skin, causing potential safety hazard risk for patients. Meanwhile, the microchannels generated by solid MNs are undergoing dynamic recovery, which is easy to cause the inaccurate drug dose.

#### Coated MNs

Coated MNs are prepared by coating drug formulation onto the surface of MNs [30]. After inserting the coated MN arrays (MAs) into skin, the drug formulation on the interface will dissolve and deeply penetrate from skin to tissue [31]. Coated MNs have also been used for the rapid transdermal delivery of macromolecules [32]. Kapoor et al. [33] coated macromolecular potent drug peptides on the surface of MNs prepared from medical-grade stainless steel liquid crystal polymers (LCPs) to achieve efficient delivery of macromolecular drugs. Preparation methods of drug-coated MNs mainly include impregnation, roller coating, layer coating, and spray coating. Impregnation method is the most commonly used approach to prepare drug-coated MNs due of its simple operation and low cost. Compared with the solid MNs, the coated MNs can simplify the drug delivery process, but the amount of drug loading capacity is limited by the specific surface area of MNs.

#### Hollow MNs

Hollow MNs, which resemble microsyringes, deliver specific drugs into skin by injecting fluid formulations into deep tissue through multiple needles at the same time [34]. Continuous delivery of molecules into skin through hollow MNs can be achieved in a variety of approaches [35]. Such systems are able to deliver larger amount of drug substances than solid, coated, and dissolving MNs [36]. Hollow MNs are made up of diverse materials, including silicon and metals, glass, polymers, and ceramics [37]. The hollow MNs can also be used for minimally invasive blood collection due to its sharp enough tips and hollow structure [38]. In addition, rapid drug delivery and controlled rate can be realized by connecting to a driving device such as syringe or micropump. However, hollow MNs require stronger mechanical strength and higher manufacturing technology. The main production methods include laser micromachining, deep ion etching reaction, deep X-ray photocopying, and wet chemical etching technology.

#### Dissolving MNs

Dissolving MNs are made by micromolding water-soluble biodegradable, biocompatible polymer, or polysaccharides to enclose drugs in MNs [39]. After insertion into the skin, the MN tip dissolves upon contact with the skin interstitial fluid (ISF) and the loaded drug molecules are then released locally over time [40]. The release kinetics of the drug depend on the dissolution rate of the constituent polymers. Therefore, controllable drug delivery can be achieved by tuning the polymer composition of the MNs or by modifying the MN fabrication process [41]. Biodegradable MNs prepared from biodegradable polymers also belong to dissolvable MNs. Once inserted into the skin, they release the loaded drug molecules by degrading rather than dissolving [42]. Compared with other types of MNs, dissolving MNs have simple preparation method, abundant ingredient materials, low risk of cross infection, and no sharp waste left in skin, which solve the problem of silicon/ceramic fracture, where needles remain inside skin that is difficult to deal with. Furthermore, it improves the drug loading on MNs to a certain extent and expands the application range of MNs.

#### Hydrogel-forming MNs

HFMNs were first reported in 2012 and typically consist of swellable polymers (crosslinked hydrogels) [43]. They are prepared from hydrogel polymers, and the processing steps are similar to dissolving MNs. HFMNs have different working mechanism from the other sorts of MNs mentioned above. When HFMNs are inserted into skin, the needle tips rapidly absorb ISF from tissue, creating continuous unblocked pores in hydrogel, and the drug penetrates and diffuses from the needle tips into the skin tissue through the tissue fluid [44]. Owing to the hydrophilic nature of hydrogels, liquid is readily absorbed, allowing the suitability for biomedical applications of ISF extraction [45]. HFMNs can resist the closure of skin pores to a certain extent and can be removed completely after being inserted into the skin. In addition, the drug release rate can be controlled by adjusting the crosslinking density of the hydrogel fibers.

### Preparation of MNs

#### Lithography

**Fig. 3.**
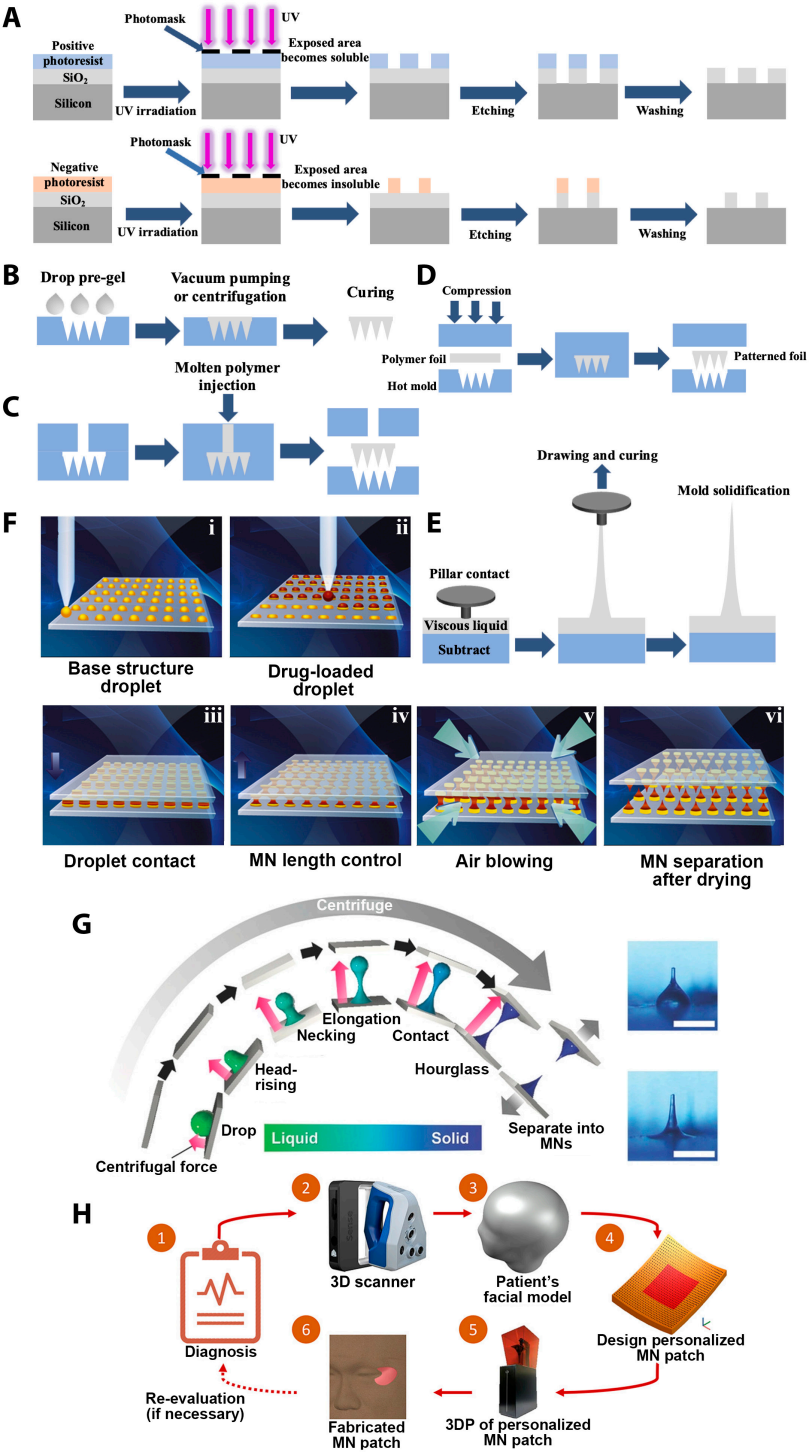
Schematic illustrations of the preparation of MNs. (A) Lithography. (B) Cast micromolding. (C) Injection molding. (D) Hot embossing. (E) Drawing lithography. (F) DAB. Reprinted with permission from [66]. Copyright 2013 Elsevier. (G) CL. Reprinted with permission from [68]. Copyright 2017 John Wiley & Sons. (H) 3D printing. Reprinted with permission from [76]. Copyright 2021 Elsevier.

Microelectromechanical system (MEMS) technology is a common method to fabricate silicon MNs [46]. First, SiO_2_ is deposited on the Si wafer, the photosensitizer is coated on the SiO_2_ surface, ultraviolet (UV) light is irradiated on the photosensitizer through a mask to form a microstructure, and then the fine microstructure preparation of the Si surface is realized by etching and removing the photosensitizer (Fig. 3A). Wang et al. [47] proposed 3 parallel subtractive manufacturing MN processes based on MEMS technology: planar pattern-to-cross-section technology process, silicon wet etching combined with UV-Photolithography, Galvanogormung, Abformung (LIGA) process, and tilt spin lithography process. All 3 methods have good reproducibility and accuracy.

#### Cast micromolding

Cast micromolding is the MN forming process by filling a solution into a pre-prepared MN mold before solidification (Fig. 3B) [48]. External force is applied by means of centrifugation or vacuum negative pressure, which ensures the solution completely filling the mold and accordingly forming the MN morphology [49]. Both dissolving MNs and HFMNs are usually prepared by cast micromolding methods [50]. Such process is suitable not only for conventional polymers and silicones but also for inorganic materials such as ceramics, aluminosilicates, and so on. Gholami et al. [51] prepared the porous alumina MN with alumina suspension as a matrix by casting the micromolding process. Alumina MN has good biocompatibility and mechanical properties and not only can accurately extract glucose from skin model but also can effectively release insulin to achieve its rapid release.

#### Injection molding

Injection molding is a molding method to form MN structures by injecting polymer solutions into molds (Fig. 3C) [52]. Medical-grade LCP can prepare MNs by injection molding, and then the analgesic drug lidocaine is coated on MNs by dip coating method to achieve long-lasting local painless effect [53]. Poly(lactic-co-glycolic acid) (PLGA) is a degradable polymer with good biocompatibility [54]. PLGA MN patches for TDD with good mechanical properties can be fabricated by injection molding process [55]. Polycarbonate (PC) is also a commonly used biocompatible polymer [56]. By changing the structure of the mold, semi-hollow and bird's beak-like PC-MNs can be prepared by injection molding to improve the complexity of solid MN application and the insufficient drug loading of coated MNs, respectively [57].

#### Hot embossing

Hot embossing forms MNs by melting the polymer into a liquid with heat and then applying pressure to fill in the mold (Fig. 3D) [58]. Poly-ε-caprolactone (PCL) is a biodegradable polymer with low melting point and suitable for hot embossing method [59]. The furosemide-loaded PCL MNs prepared by hot embossing method is suitable for effective drug release [60]. Li et al. [61] fabricated the PLGA gradient porous microneedle arrays (GPMAs) for drug delivery using a modified thermal embossing process. The MNs with gradient pore distribution were formed by the modulation of gradient thermal field and pressure. The fluorescent dye Rhodamine B was loaded into GPMA, and it was observed that the fluorescence intensity inside GPMA gradually decreased from the tip to end, indicating that it has a gradient porosity. Loading insulin into GPMA achieved an evident therapeutic effect on diabetic mice.

#### Drawing lithography

The drawing lithography method is to coat a negative photoresist on a glass substrate, the photoresist is stretched into a needle-like structure as a solid MN mold, a metal coating is formed on the surface of MN mold by electroplating, and then the photoresist was removed to obtain hollow metal MNs (Fig. 3E) [62]. By controlling the thickness of the SU-8 photoresist coating to tune the inner diameter of the MNs, Lee et al. [63] used tensile lithography to fabricate a minimally invasive blood collection through a length of 1,800 μm and an inner diameter of 30 or 60 μm metal hollow MNs with ultrahigh aspect ratio for minimally invasive blood collection. Chen et al. [64] adopted a novel magnetorheological drawing lithography (MRDL) method for MN fabrication. Curable magnetorheological fluid replaces the role of photoresist and is directly drawn from the substrate into needles and cured under an external magnetic field, which is simpler and more convenient than traditional drawing lithography. On this basis, Chen et al. [65] prepared a flexible resin microneedle array (FMA) by MRDL and then used FMA as the master template to prepare dissolvable MNs by micromolding, realizing the TDD.

#### DAB and CL

DAB and CL both obtain MNs by forming hourglass-shaped droplets between 2 parallel substrates before solidification. The DAB method uses the movement of parallel substrates to connect viscous droplets between the upper and lower substrates, and directly blows the polymer solution through air to solidify it to form MNs (Fig. 3F) [66]. Since no external conditions such as heating or UV curing and molds are required, DAB possesses the merits of mild molding conditions and simple and rapid preparation, and the prepared MNs become more conducive for loading the external environment-sensitive drugs. Park et al. [67] employed DAB to load house dust mites into hyaluronic acid (HA) MNs for transdermal immunotherapy of asthma. Compared with traditional subcutaneous immunotherapy, it has the advantages of less allergic reaction and high drug compliance.

CL uses centrifugal method to elongate the polymer droplets on the surface of the lower substrate under centrifugal force (Fig. 3G). After contacting with the upper substrate, the droplets form an hourglass-like shape under the action of self-plasticity, resulting in centrifugal evaporation and an hourglass-like structure, gradually thinned and finally solidified to obtain MAs [68]. Compared with DAB, CL does not require additional stretching equipment and has the advantages of mild preparation conditions, and its operational process is also simpler. Huh et al. [69] compared DAB with the CL method by preparing MNs loaded with epidermal growth factor and ascorbic acid followed by analyzing the drug activity. Due to the lower pressure during the fabrication process, CL can more effectively maintain the activity of encapsulated drugs and is more suitable for the loading of relatively fragile drugs in MNs. Subsequently, a high dose of atopic dermatitis (AD) drug triamcinolone (TA) was dissolved in polyvinylpyrrolidone (PVP) and HA to form a viscous liquid, and the TA-MN patch was successfully prepared by the CL method [70]. Owing to the high drug loading, the drug dose of the TA-MN patch applied once is equivalent to TA injections and TA cream application twice a day, showing an excellent therapeutic effect on AD.

#### 3D printing

3D printing, also known as additive manufacturing, can rapidly, accurately, and stably prepare materials with fine structure, and as an emerging technology, it can facilitate the development of material and biomedical science (Fig. 3H) [71]. The micrometer-scale structures of MNs can be 3D-printed, which can not only customize structures to improve the performance of MNs but also greatly enhance the efficiency of MN manufacturing, affording mass production. Stereolithography appearance (SLA) 3D printing technology prepares designed models by layer-by-layer photocuring of UV-sensitive polymers [72]. Economidou et al. [73] designed the MN structure through SolidWorks software, prepared a biocompatible Dental SG resin MN patch by SLA, and coated insulin on the surface of MNs by inkjet printing technology to achieve the efficacy of diabetes treatment. Then, the good biocompatible polychlorolactose-polyvinyl acetate-polyethylene glycol was used as substrate material to prepare MNs, and the cisplatin antitumor drug coating on the surface of MNs was prepared by inkjet printing technology to obtain antitumor ability [74]. Digital light processing (DLP) 3D printing technology projects the image of the cross section of the object onto the photosensitive liquid resin through the DLP chip to induce solidification and move it layer by layer to complete the preparation, which has the advantages of fast printing speed and high resolution [75]. Lim et al. [76] used DLP to print a biocompatible polymer blended with polyethylene glycol diacrylate (PEGDA) and vinylpyrrolidone (VP) into a customized MN patch, which has anti-wrinkle function by loading AHP-3 small peptides to achieve anti-aging effects. Two-photon polymerization (2PP) 3D printing technology realizes the selective polymerization of photosensitive resins by ultrashort laser pulses from a NIR femtosecond laser source, which displays the characteristics of geometric control and scalable resolution [77]. Cordeiro et al. [78] printed multiple types of MA molds by 2PP to optimize the morphology design for drug delivery patches. Szeto et al. [79] prepared hollow MNs that could be inhaled in guinea pigs' perilymph through 2PP technology. Proteomic analysis by collecting perilymph through MNs avoided the damage of cochlear tissue and provided a new horizon for the diagnosis of human inner ear.

## Applications of MNs

### Disease treatment

#### Wound healing

##### Transdermal delivery of active ingredients

**Fig. 4.**
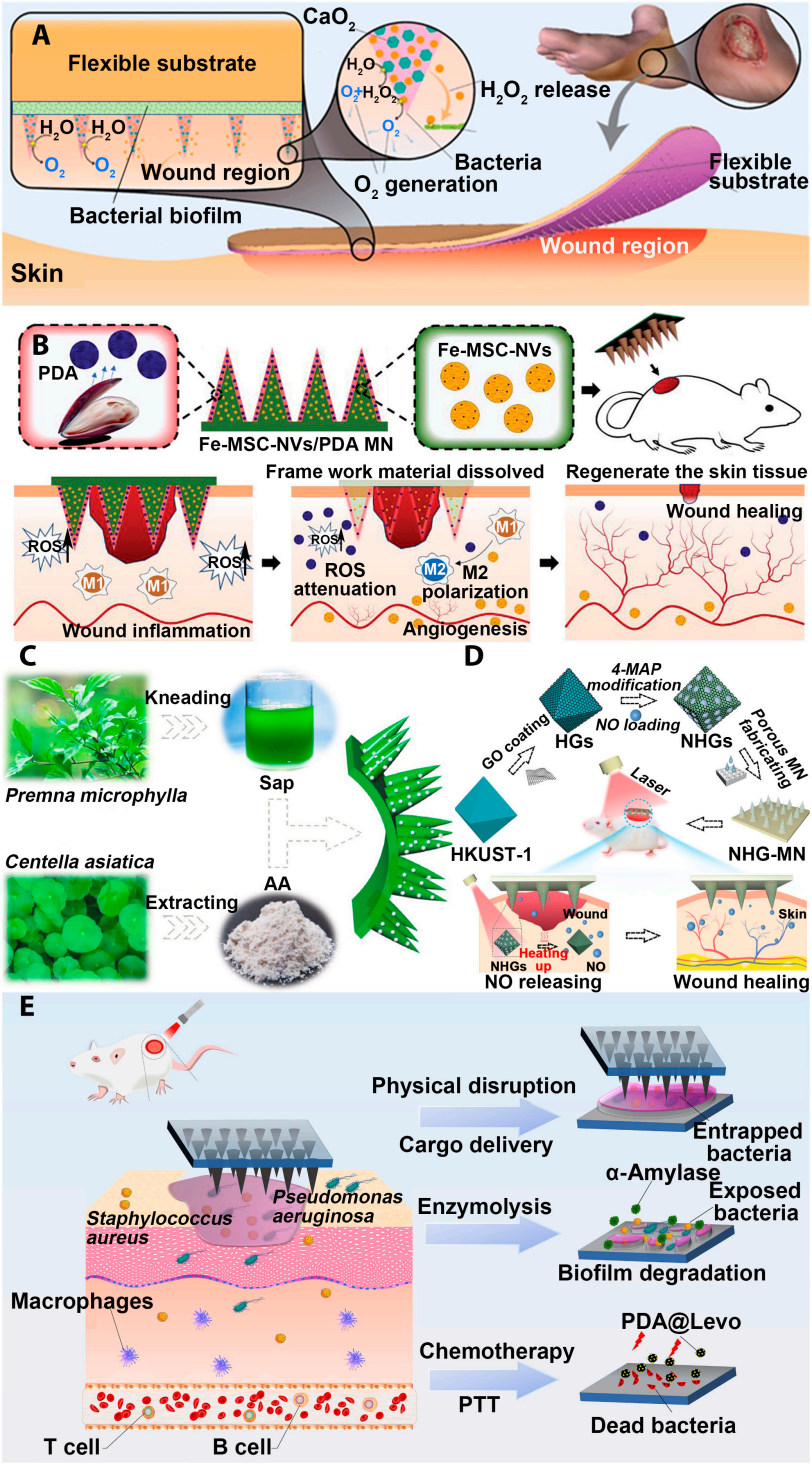
MNT for wound healing. (A) Calcium peroxide (CaO_2_)-loaded PVP MN patch for chronic nonhealing wounds. Reprinted with permission from [81]. Copyright 2021 American Chemical Society. (B) Fe-MSC artificial nanovesicles/PDA shell with core/shell structured MeHA MN patch for chronic diabetic wound healing. Reprinted with permission from [88]. Copyright 2022 John Wiley & Sons. (C) AA loaded pure CHMN for wound healing. Reprinted with permission from [90]. Copyright 2021 Elsevier. (D) PEGDA MN patches loaded with GO-coated metal organic framework (MOF) and nitric oxide (NO) molecules for photothermal/nitric oxide combined therapy for promoting wound healing. Reprinted with permission from [106]. Copyright 2022 John Wiley & Sons. (E) PVA MNs loaded with levofloxacin-encapsulated PDA NPs and α-amylase for antibiotic/enzymolysis/photothermal triple therapy to promote wound healing. Reprinted with permission from [113]. Copyright 2022 Elsevier.

**Fig. 5.**
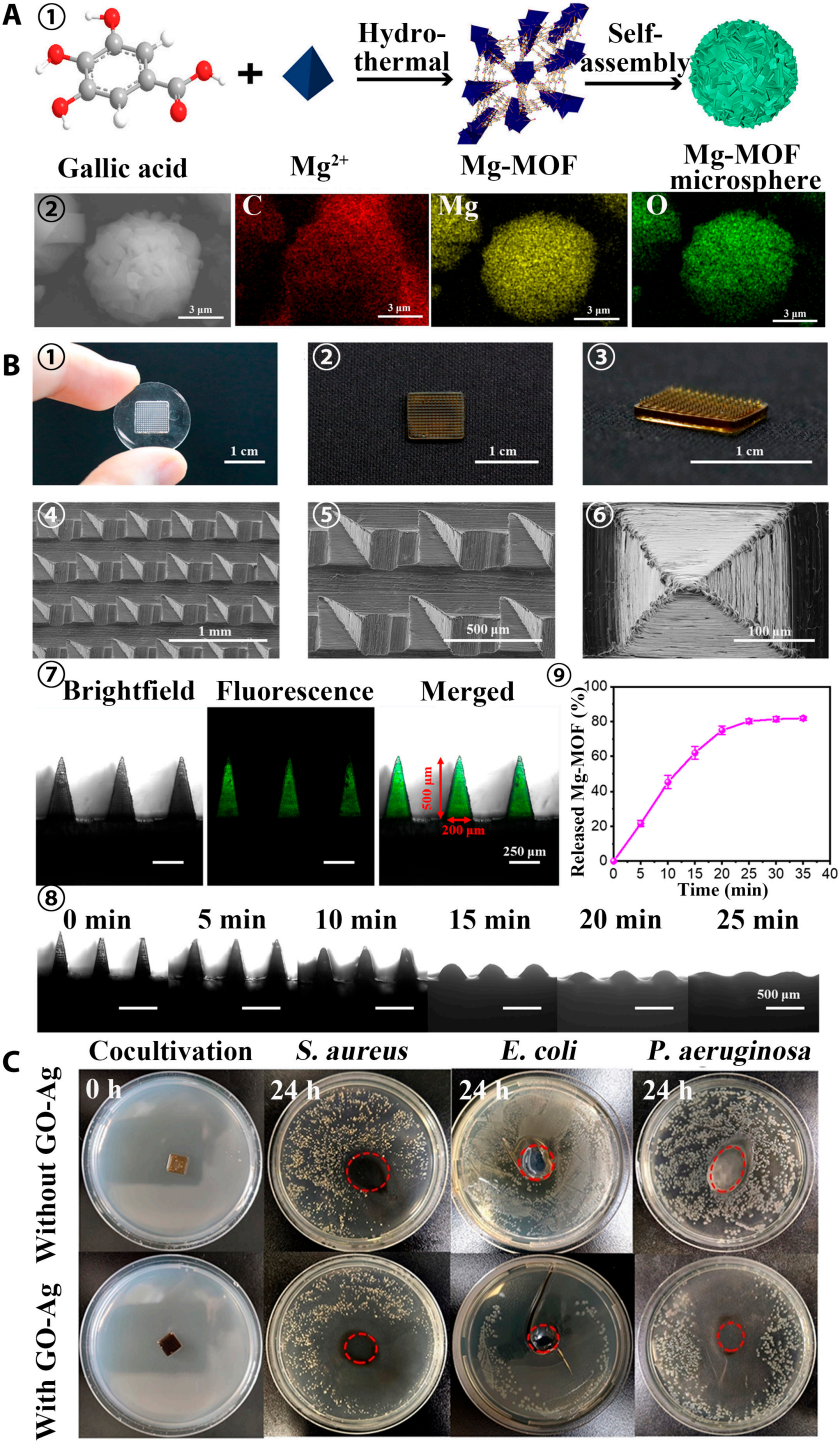
MOF-GO-Ag-MN patches for wound healing. (A) Synthesis and characterization of Mg-MOF. (1) Schematic illustration of the synthesis of Mg-MOF microsphere. (2) Scanning electron microscopy (SEM) image and elemental mapping of Mg-MOF. (B) Synthesis and characterization of MN-MOF-GO-Ag. (1) Photographic image of PDMS MN mold. (2 and 3) Top-down and isometric photographic images of an MN-MOF-GO-Ag. (4 to 6) SEM images of MN-MOF-GO-Ag. (7) Fluorescence microscopic images of the tips of fluorescein isothiocyanate (FITC)/bovine serum albumin (BSA)-loaded MN-MOF-GO-Ag. The labels revealed the detailed dimensions of the MN tips: a base diameter of 200 μm and a height of 500 μm. (8) Brightfield morphologic images of MN-MOF-GO-Ag after moisture absorption at different time points (75% humidity box, room temperature). (9) Percentages of the released Mg-MOF from an MNMOF-GO-Ag over time in the presence of phosphate-buffered saline (PBS). (C) In vitro antibacterial capabilities of MN-MOF-GO-Ag. Representative photographic images of different bacteria strains treated with MN patches with or without GO-Ag. The red dotted circles indicate the position of MN patches. Reprinted with permission from [84]. Copyright 2021 American Chemical Society.

**Fig. 6.**
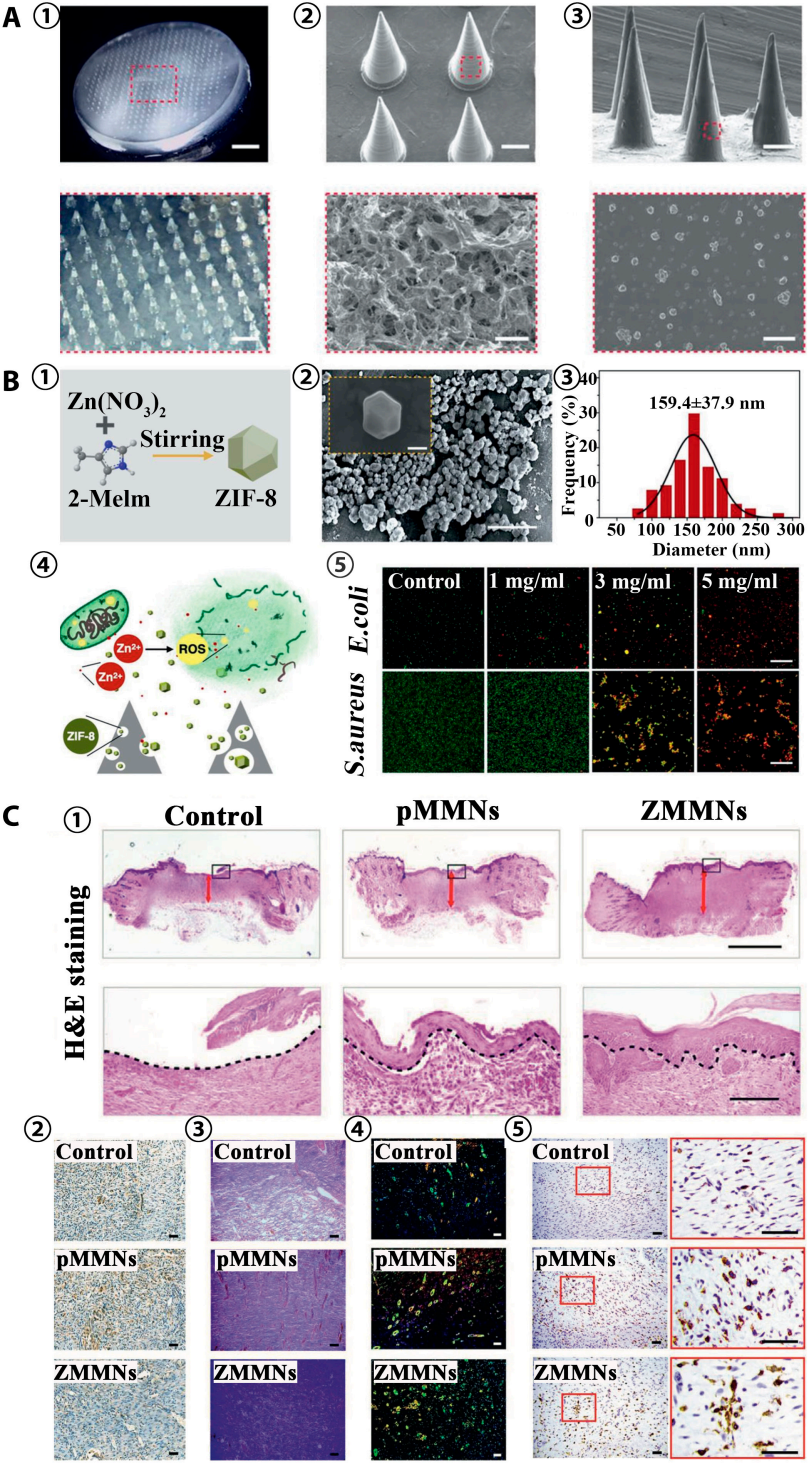
Zn-MOF MN patches for wound healing. (A) Characterization of ZIF-8@MeHA-MNs. (1) Optical images. (2) SEM images of the surface structure. (3) SEM images of ZIF-8 NPs encapsulated in the MNs. (B) ZIF-8 NP characterization and antibacterial property. (1) Schematic diagram of ZIF-8 synthesis process. (2) Aggregation and individual SEM images of ZIF-8 NPs. (3) Diameter distribution of ZIF-8 NPs. (4) Schematic of the antibacterial effect of ZIF-8 NPs. (5) Live and dead bacteria staining of *E. coli* and *S. aureus* cocultured with different ZIF-8 concentrations. Scale bars are 1 μm in (2), 100 nm in the insert image, and 100 μm in (5). (C) Evaluation of the MNs on wound healing. (1) Optical images and the corresponding amplified images of H&E staining. (2) Immunohistochemistry staining of IL-6. (3) Collagen deposition images of Masson's trichrome staining (MTC). (4) Double immunofluorescent staining of CD31 (red) and 𝛼-smooth muscle actin (𝛼-SMA) (green) for vascularization analysis. (5) Immunohistochemistry staining of CD163 for M2 macrophage analysis. Scale bars are 100 μm in (1) and 50 μm in (2) to (4). Experiments are carried out in triplicate. Reprinted with permission from [86]. Copyright 2021 John Wiley & Sons.

Active ingredients can be effectively delivered to the lesion site through MNs, achieving efficient delivery and better therapeutic effects. Chronic nonhealing wounds are colonized by bacteria that often develop into biofilms and act as a physicochemical barrier to internal bacteria, inducing chronic inflammation and tissue hypoxia [80]. Removing biofilms not only requires repeated treatments but also can be painful. Woodhouse et al. [81] loaded calcium peroxide into PVP MNs for painless removal of bacterial biofilms (Fig. 4A). The MNs could effectively kill gram-positive bacteria and gram-negative bacteria, provide oxygen channels to ameliorate tissue hypoxia, and had good cytocompatibility. The wound healing process can generally be divided into 3 stages: inflammation, proliferation, and tissue remodeling [82]. Therefore, treatments with multifunctions of anti-inflammatory, boosting cell proliferation, and tissue remodeling can greatly improve the wound healing effect. Some metal ions can both inhibit bacterial infection and contribute to cell proliferation for tissue regeneration; hence, they serve as essential substances for basic biochemical reactions [83]. Yin et al. [84] separately loaded multifunctional magnesium-organic frameworks (Mg-MOFs) and graphene oxide-silver nanocomposites (GO-Ag) into poly-γ-glutamic acid (γ-PGA) hydrogels as MN tips and backing base to prepare MOF-GO-Ag-MN patches (Fig. 5). In vitro antioxidant experiments showed that MOF-GO-Ag-MN could release Mg-MOF with good antioxidant effect, facilitate the proliferation of human dermal fibroblasts after oxidative stress by scavenging the cytotoxic ROS, and have excellent antioxidant performance. The in vitro antibacterial experiments confirmed that MOF-GO-Ag-MN could inhibit the growth of *S. aureus*, *Escherichia coli*, or *Pseudomonas aeruginosa*. The migration of human umbilical vein endothelial cells (HUVECs) was tested by in vitro cell migration experiments, and MOF-GO-Ag-MN had the best effect of promoting cell migration. The wound healing in diabetic mice was evaluated, and it was found that MOF-GO-Ag-MN treatment had the smallest wound area, the largest tissue regeneration, and the fastest new angiogenesis rate, demonstrating the best wound healing effect. Zn-MOFs can eliminate bacteria by releasing zinc ions and have good biocompatibility and antibacterial properties [85]. The Zn-MOF MN patches prepared by encapsulating Zn-MOFs into degradable methacrylated HA (MeHA) hydrogels enabled sustained release of active ingredients in the wound area (Fig. 6) [86]. The Zn-MOF MN patch not only possesses excellent antibacterial activity but also significantly accelerates epithelial tissue regeneration and neovascularization, thereby promoting wound healing.

Chronic diabetic wounds can induce skin ulcers and impaired angiogenesis, ROS overexpression, and persistent inflammation, which make wounds difficult to heal [87]. Therefore, it is desirable to have a multifunction MN patch with antioxidant, anti-inflammatory, and accelerating angiogenesis to promote diabetic wound healing. Ma et al. [88] encapsulated iron nanoparticle (NP)-treated mesenchymal stem cell (MSC)-derived artificial nanovesicles (Fe-MSC-NVs) into the inner HA of MN tips with polydopamine NPs (PDA NPs). MeHA was used as the shell of the MN tips to prepare Fe-MSC-NVs/PDA MNs with core-shell structure (Fig. 4B). PDA NPs could protect skin tissue from damage by scavenging excessive ROS generated in the inflammatory response, while Fe-MSC-NVs had the ability to facilitate HUVEC migration and proliferation to promote angiogenesis and assess macrophage polarization from a pro-inflammatory M1 phenotype to an anti-inflammatory M2 phenotype. PDA NPs could be slowly released from Fe-MSC-NVs/PDA MNs with sustained antioxidant effects. In vivo diabetic wound healing experiments showed that the Fe-MSC-NVs/PDA MN group had the smallest wound area, and the wound was basically completely healed after 12 d of treatment, displaying great potential for wound healing.

In addition to the synthetic active ingredients, traditional Chinese medicine is a time-honored, ancient, and unique drug system, which has achieved considerable therapeutic effects from China to all over the world [89]. Combining MNs with traditional Chinese medicine not only overcomes the complex operation difficulty of its medication process but also greatly improves the delivery efficiency of traditional Chinese medicine to achieve better therapeutic effects. Chi et al. [90] prepared a pure Chinese herbal MN patch (CHMN) via saline-induced coagulation of 2 traditional Chinese herbs, *Premna microphylla* and *Centella asiatica*, and loaded asiatic acid (AA) as a pharmaceutical active ingredient into MNs (Fig. 4C). In vitro cell experiments showed that CHMN did not affect the normal proliferation of mouse embryonic fibroblast cells (NIH-3T3), had no cytotoxicity, and showed good cytocompatibility. The in vitro antibacterial experiments using CHMN could effectively kill *E. coli* and *S. aureus* and had excellent antibacterial ability. CHMN was applied to rat wounds, and it was observed that the wound healing effect of CHMN group was the best during the 9-d healing process. Hematoxylin and eosin (H&E) staining discovered that the thickness of regenerated granulation tissue in CHMN group was the largest. Immunohistochemical analysis of the expression of the pro-inflammatory factor interleukin-6 (IL-6) in the wound area discovered that the CHMN group had the lowest IL-6 expression. Masson's trichrome staining showed that the CHMN group had the most collagen deposition, and immunofluorescence staining showed that the wound site of the CHMN group formed more new blood vessels. These results substantiate that CHMN has excellent ability to promote wound healing and has valuable application potential. Chitosan is a derivative of chitin, which not only has good biocompatibility but also has antibacterial effect. Using chitosan as the matrix of MNs can enable the antibacterial function [91]. Wound healing-promoting chitosan MN array (CSMNA) patches were fabricated from temperature-responsive poly(N-isopropylacrylamide) (pNIPAM) hydrogels encapsulating vascular endothelial growth factor (VEGF) in combination with chitosan [92]. Controlled release of VEGF could be achieved by utilizing the temperature-responsive properties of pNIPAM. The 99% mortality of *S. aureus* and *E. coli* cocultured with the CSMNA patch indicated its excellent antibacterial performance. In vivo animal experiments in rats showed that CSMNA patch could significantly promote wound healing by favoring the granulation tissue formation and collagen deposition, inhibiting the expression of pro-inflammatory factors, and accelerating angiogenesis, with high potential for practical applications.

##### Photothermal therapy

PTT is a treatment method in which photothermal agents can convert light energy into heat under NIR laser irradiation to kill bacteria or pathogens [93]. Adjusting the laser power, photosensitizer concentration and irradiation time can achieve a controllable hyperthermia effect [94]. Compared to traditional antibiotic therapy, PTT has the advantage of circumventing drug resistance and causing minimal thermal damage to normal living tissue [95]. A moist wound environment can promote the release of growth factors to promote wound healing, so maintaining an appropriate moisture content in the wound area is beneficial to promote wound healing [96]. Sun et al. [97] used soluble polyvinyl alcohol (PVA) as the matrix for MNs and metal-organic framework (MOF)-derived multifunctional porphyrin-like metal-centered NPs (PMCS) with photothermal and nano-enzyme properties as the support to prepare PMCS@MN patch with Band-Aid performance. Under the irradiation of 808-nm laser, the PMCS@MN patch produced local high temperature, PVP was dissolved and adhered to the wound surface to maintain moisture, and PMCS was released to generate ROS to eliminate bacteria. The results of in vitro antibacterial tests and in vivo animal tests both verified that PMCS@MN showed high performance to promote wound healing.

##### Photodynamic therapy

**Fig. 7.**
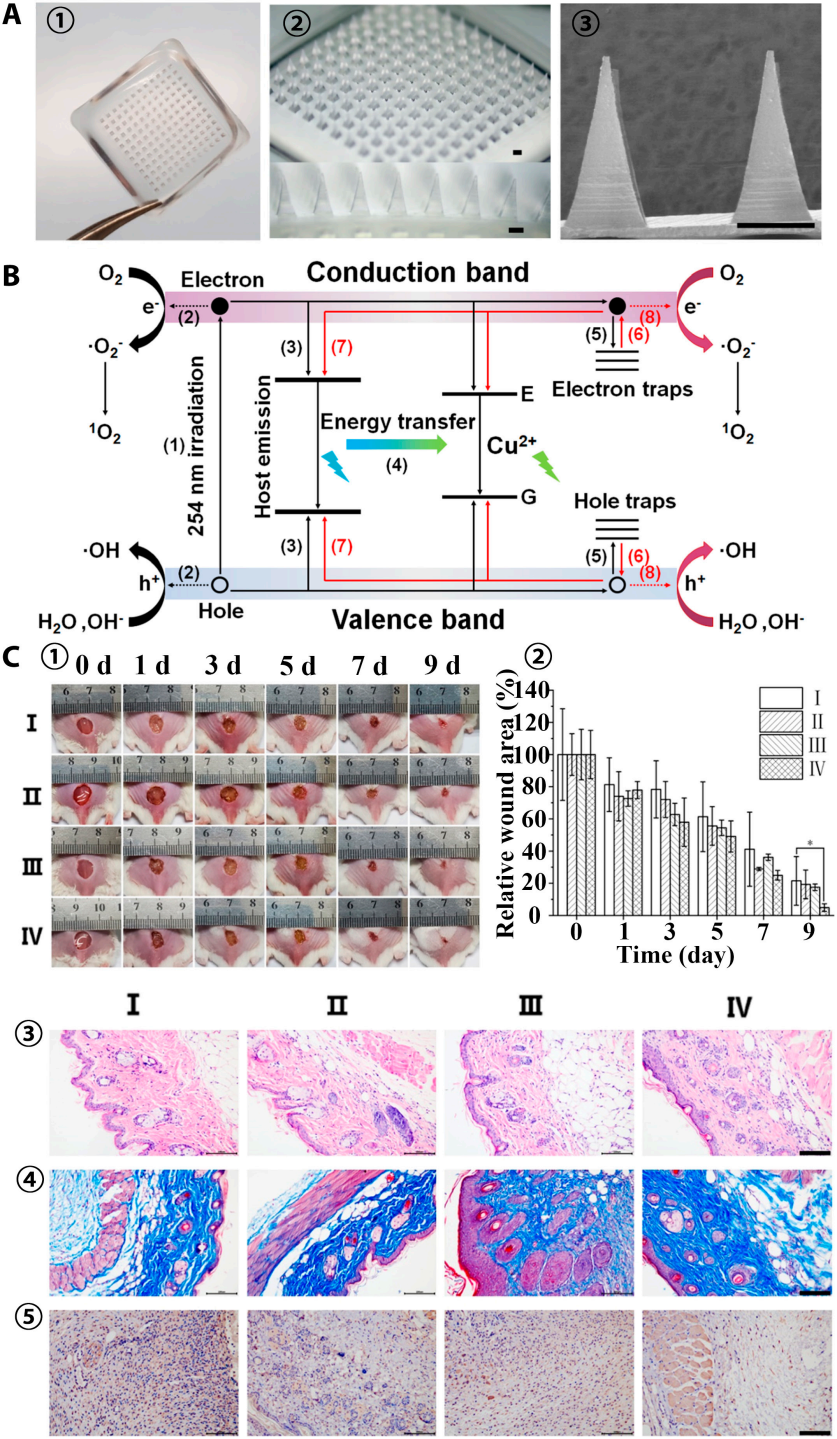
ZGC@MN patches for PDT wound healing. (A) (1) Representative photograph of the as-prepared ZGC@MN patch. (2) Microscopy images of ZGC@MNs. Scale bars are 300 μm. (3) SEM image of the tips in ZGC@MNs. Scale bar is 300 μm. (B) Possible mechanism of the processes for persistent luminescence and the constant ROS production of ZGC after stopping excitation. (C) (1) Representative photographs of the skin wounds of different groups. (2) Relative wound area of different groups at different time points during the healing process. (3) H&E staining, (4) Masson trichrome staining, and (5) immunohistochemistry staining of TNF-α of the wounds on day 9. All the scale bars are 100 μm. (I) No treatment, (II) blank MNs, (III) ZGC@MNs, and (IV) ZGC@MNs with pre-illumination. Reprinted with permission from [103]. Copyright 2022 American Chemical Society.

PDT by accumulating photosensitizers around target cells can generate cytotoxic ROS (e.g., singlet oxygen ^1^O_2_, superoxide anion O_2_^−^, and hydroxyl radical OH^•^) under light so as to eliminate target cells, and since no drug is used, no drug resistance occurs [98]. The MNs loaded with photosensitizer methylene blue can effectively kill *S. aureus* and *E. coli* with high antibacterial activity [99]. The conventional photosensitizers require constant light exposure, may cause skin damage, and largely depend on oxygen to engender ROS. But oxygen helps to promote cell proliferation and tissue remodeling at the wound site, and rapid consumption of oxygen creates a hypoxic environment that is not beneficial to infectious wound healing [100,101]. Persistent luminescent nanomaterials (PLNMs) can continue to emit light for hours to days after stopping excitation, and the long-term persistent photocatalytic effect (LPPC) can be a continuous generation of ROS [102]. Gong et al. [103] synthesized a PLNM material Cu^2+^ doped Zn_2_GeO_4_ (ZGC) and combined it with HA MN to prepare ZGC@MN patches that promote wound healing (Fig. 7). Just irradiated by 254-nm UV light for 1 hour, the ZGC can continue to emit light for 24 hours, continuously producing ROS, and has prominent stability at neutral pH, but can be degraded at acidic pH. The ZGC@MN can effectively kill bacteria, promote collagen deposition and angiogenesis in wound tissue, and inhibit the expression of inflammatory factors to effectively promote wound healing.

##### Alliance therapy

In general, it is difficult for a single therapy to achieve the best curative efficacy; hence, taking multiple therapies at the same time for combination therapy can obviously improve the treatment effect [104]. Generating gas that is beneficial for wound healing together with PTT can effectively promote wound healing. Nitric oxide (NO) is usually involved in comprehensive physiological and pathological processes as an endogenous gas molecule and has various functions such as vasodilation, angiogenesis, infection elimination, immune regulation, signal transmission, and integration [105]. Yao et al. [106] fabricated a PEGDA MN patch with NIR photothermal responsive NO controllable release by coating GO shell layer on MOFs and loading NO molecules into it, realizing the combination therapy of NO molecules and PTT, which could promote the healing of diabetic wounds and possess broad application prospects (Fig. 4D). Ma et al. [107] employed GO as photothermal agent, S-nitroglutathione (GSNO) as NO donor, and PVA as MN matrix to prepare GSNO-HFMNs with photothermal controllable release of NO. This favors tissue regeneration and hinders bacteria growth while expediting the healing process of biofilm-infected wounds.

Oxygen maintains normal cell growth and respiration, favorably assisting to promote tissue generation and recovery [108]. Combining oxygen carriers with MN can enable oxygen release into wound to improve healing efficacy. Black phosphorus (BP) has prominent photothermal properties and biocompatibility, thus fairly potential for biomedical applications [109]. Zhang et al. [110] loaded BP quantum dots and oxygen-enriched hemoglobin (Hb) into GelMA MN tips to fabricate BP-Hb MNs with photothermally responsive oxygen controllable release. The increment of temperature reduces the oxygen binding capacity of Hb to facilitate the release of oxygen, and the controllable release of oxygen is achieved by adjusting the temperature by photothermal approach. Through wound healing experiments in diabetic rats, it was found that the BP-Hb MN patch group had the highest wound closure rate, the thickest regenerated epithelial tissue, the largest amount of collagen deposition, the least secretion of IL-6 inflammatory factors, and the highest wound vascular density, indicating that it has optimal performance to promote wound healing ability.

Exopolysaccharide is one of the important components of bacterial biofilm [111]. Enzymatic degradation of exopolysaccharide to disintegrate the bacterial biofilm can effectively eradicate bacteria [112]. Yu et al. [113] loaded the antibiotic levofloxacin into PDA NPs (PDA@Levo NPs) and encapsulated into PVA MN together with α-amylase to prepare a wound-healing-promoting MN patch with triple therapy consisting of antibiotic, enzymatic hydrolysis, and photothermal treatment (Fig. 4E). Both in vitro and in vivo anti-biofilm experiments showed that the MN patch could effectively penetrate the biofilm and release PDA@Levo NPs. At the same time, applying PTT could decompose the biomass of the biofilm and kill the bacteria efficiently to achieve excellent antibacterial effect. In vivo experiments in rats showed that the MN patch achieved the optimized wound healing effect by eliminating biofilms in the wound, reducing neutrophil infiltration, promoting collagen deposition, facilitating angiogenesis, and inhibiting the expression of inflammatory factors. The above results fully indicate that MNT has great potential for practical applications in the field of wound healing.

#### Tumor treatment

##### Chemotherapy

**Fig. 8.**
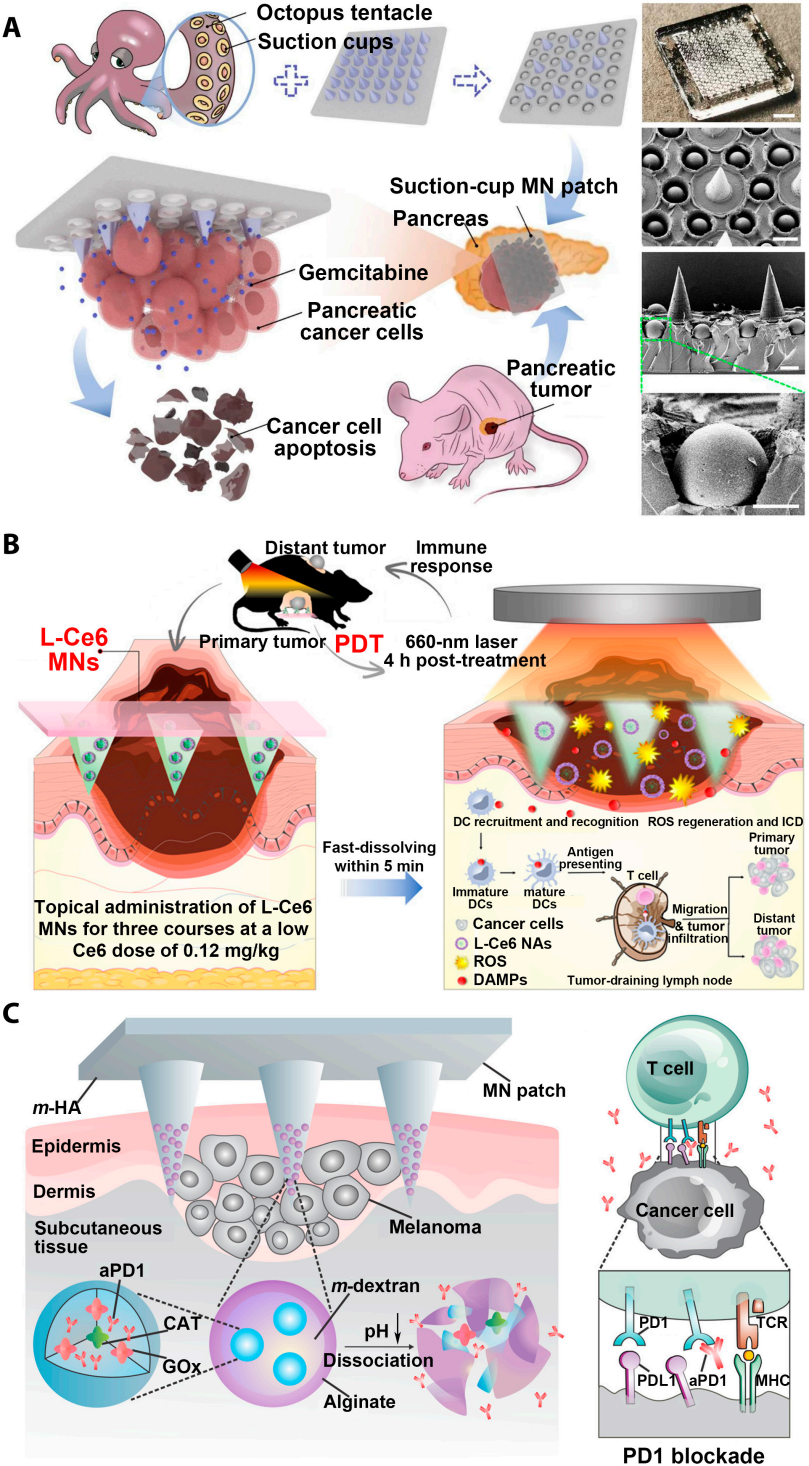
MNT for tumor treatment. (A) Gemcitabine-loaded octopus sucker-structured GelMA MN patch for pancreatic cancer chemotherapy. Digital image and SEM images showed the MN patch with octopus tentacle-mimicked structure for better adhesion ability, which enabled sustained drug release at the tumor site. Reprinted with permission from [117]. Copyright 2022 Elsevier. (B) Low-dose L-Ce6 photosensitizer-loaded oligo-HA MN for tumor PDT. L-Ce6 NAs could be successfully delivered to the tumor site by MN, and the tumor was ablated by ROS generated by PDT. Subsequently, antitumor immune responses were activated by MNs triggering immunogenic cell death (ICDs) and releasing danger-associated molecular patterns (DAMPs), thereby increasing T cell infiltration in bilateral tumors and inhibiting the growth of distant tumors. Reprinted with permission from [125]. Copyright 2021 American Chemical Society. (C) HA MN loaded with anti-PD-1 antibody (aPD-1) and glucose oxidase (GOx) for tumor immunotherapy. The GOx/CAT enzyme system was immobilized in the NPs, and aPD-1 was effectively released through the continuous dissociation of the NPs caused by the enzyme-mediated conversion of glucose to gluconic acid. The immune system was activated by the blockade of PD-1 by aPD-1 to eliminate skin cancer cells. Reprinted with permission from [128]. Copyright 2016 American Chemical Society.

Chemotherapy is one of the most commonly used therapeutic strategies for tumor treatment, but due to the lack of targeting, not only it is difficult to achieve the best therapeutic effect but also it produces serious side effects [114]. Pancreatic cancer (PC) is one of the most lethal malignancies in the world. Due to its difficulty in detection, most patients are diagnosed at a late stage, which is difficult to undergo surgery and is usually treated with chemotherapy [115]. Gemcitabine is used as a standard treatment drug for PC, but it is difficult to attain high drug penetration efficiency due to the dense stromal barrier of pancreatic tumors [116]. The MN patch can penetrate the stromal barrier so that the drug can be released directly into the tumor, leading to promising therapeutic effect. Fu et al. [117] loaded gemcitabine into a high-adhesion GelMA MN patch with an octopus sucker structure to achieve sustained drug release (Fig. 8A). Even facing the irregular surface of the tumor, the high-adhesion MN patch can be easily adhered and can effectively control the drug release kinetics. Because of the sustained release of the drug, in vitro cell experiments confirmed that the MN patch had a long-term inhibitory effect on the human pancreatic cancer cell line Capan-1, and in vivo animal experiments showed that the MN patch had a more significant tumor inhibitory effect than the same dose of gemcitabine injected intraperitoneally while showing excellent biocompatibility. Wang et al. [118] developed a heterogeneous silk fibroin microneedle (SMN) patch to achieve controllable release of multiple drugs, which can stop bleeding during surgery, inhibit postoperative angiogenesis, and promote apoptosis of glioblastoma cells. The biocompatibility and biodegradability of silk ensures the safety of intracranial implantation. The MN structure provides a channel for drug delivery. The combined administration of angiogenesis inhibitors and antitumor drugs effectively modulates the tumor cell microenvironment, thereby significantly suppressing tumor volume and improving survival rate of mice. The SMN patch provides a promising drug delivery system for clinical application in intracranial tumor therapy.

##### Photothermal therapy

PTT achieves antitumor effect by inactivating tumor cells by generating local hyperthermia [119]. Generally, better tumor treatment effect is achieved by co-administration of PTT and antitumor drugs [120]. Docetaxel (DTX) is a broad-spectrum antitumor drug with evident antitumor effect [121]. Antitumor MN patches with synergistic therapeutic effect of photothermal therapy and chemotherapy were prepared by simultaneously loading DTX-loaded MPEG-PDLLA-DTX micelles and photothermal agent of PEGylated gold nanorod (GNR-PEG) onto biodegradable poly(l-lactic acid) (PLLA) MN patch [122]. The MN patch can effectively inhibit the growth of A431 tumor in mice, showing strong antitumor effect. Zhao et al. [123] coated HA into MOF encapsulated with photothermal agents and chemotherapeutic drugs and combined with dissolving MN patches to prepare antitumor MNs for PTT and chemotherapy. In vivo antitumor experiments in mice showed that the synergistic effect between chemotherapy and PTT had a prominent inhibitory effect on tumors with strong antitumor effect.

##### Photodynamic therapy

PDT kills tumor cells by radiation ROS generated by photosensitizers under light and has the advantages of noninvasiveness, high selectivity, and low side effects [22]. The efficient delivery of photosensitizers to tumor sites is critical to improve the therapeutic effect of PDT, and MNs can just achieve efficient TDD. Therefore, the combination of PDT and MNs can effectively improve the therapeutic effect of superficial tumors [124]. Bian et al. [125] loaded low-dose chlorin e6 (L-Ce6) photosensitizers into oligo-HA MNs for efficient PDT therapy (Fig. 8B). L-Ce6 MN can deliver L-Ce6 to a depth of 500 μm under skin so that the drug can be efficiently and accurately delivered to the target site. The results of antitumor experiments in mice showed that PDT of L-Ce6 MN could effectively eliminate tumor cells and activate tumor immune responses to achieve prominent antitumor effects.

##### Immunotherapy

Immunotherapy has been one of the most effective methods for treating malignant tumors; circulating and expanding antitumor responses can be initiated by the immune system to effectively remove tumor cells. In other words, the immune system can be activated by tumor-specific antigens to kill tumor cells, and then more additional tumor-associated antigens are released from dying tumor cells to further expand the antitumor response, which is called the cancer immune cycle [126]. Melanoma is one of the common malignant tumors in humans, and blocking the programmed death-1 (PD-1) pathway of melanoma by immunotherapy presents an evident antitumor effect [127]. Combining MNs with immunotherapy can achieve effectively sustained delivery of immunosuppressants, leading to better therapeutic effect. Wang et al. [128] loaded dextran NPs encapsulated with anti-PD-1 antibody (aPD-1) and glucose oxidase (GOx) into HA MNs to prepare MN patch for aPD-1 immunotherapy (Fig. 8C). Blood glucose can be converted into gluconic acid by GOx in an aerobic environment, and NPs will dissociate under acidic conditions, thereby releasing the encapsulated aPD-1 to achieve a better immunotherapy and antitumor effect [129]. Encapsulating tumor vaccines into biodegradable MN patches can achieve sustained release of tumor antigens, thereby inducing long-term antitumor responses and achieving prominent antitumor effects [130].

##### Synergistic therapy

**Fig. 9.**
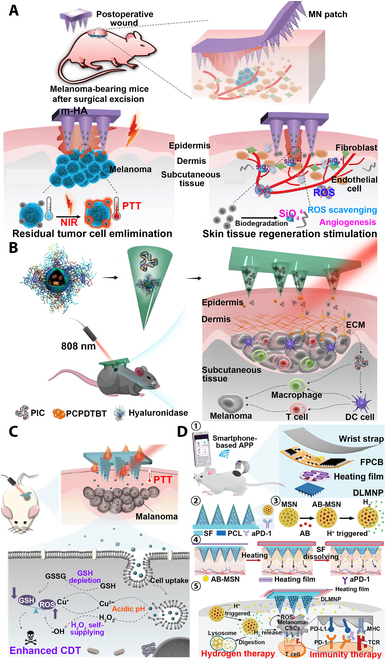
MNT for tumor synergistic treatment. (A) MeHA MN patches loaded with SiO_2_-modified melanin NPs for PTT of tumors and wound healing. CINP@SiO_2_ MN patch not only had PTT antitumor effect but also could release SiO_4_^4-^ to stimulate the proliferation and differentiation of endothelial cells and accelerate collagen deposition and re-epithelialization to promote wound recovery. Reprinted with permission from [132]. Copyright 2022 John Wiley & Sons. (B) PVP MN patch delivers hyaluronidase-modified semiconductor polymer NPs containing poly(cyclopentadithiophene-alt-benzothiadiazole) and immune adjuvant polyinosinic–polycytidylic acid (PIC) for synergistic immune/photothermal therapy. Hyaluronidase-modified photothermal-responsive semiconducting polymer NPs (HSPN) and TLR3 agonist PIC were loaded into the MN patch. Hyaluronidase could dissolve extracellular matrix (ECM) so that drugs and immune cells could penetrate deep into the tumor. Reprinted with permission from [143]. Copyright 2021 American Chemical Society. (C) CuO_2_-loaded PVP MN patch for synergistic chemodynamic/photothermal therapy of melanoma. CuO_2_ NPs in MN could release Cu^2+^ as a Fenton catalyst in the tumor microenvironment to convert hydrogen peroxide into toxic hydroxyl radicals, could remove overexpressed reducing substances [such as glutathione (GSH)] and thus enhance the therapeutic effect of CDT, and could also be used as a photothermal agent to achieve PTT. Reprinted with permission from [145]. Copyright 2022 Elsevier. (D) Controlled hydrogen and aPD-1 release silk-based MN patch for synergistic immune/hydrogen therapy of melanoma. (1) Schematic diagram of wearable silk-based MN device (SMND) treatment and composition, which mainly consisted of a double-layered MN patch (DLMNP), a heating-film, a flexible print circuit board (FPCB), a smartphone-based application (APP), and a wrist strap. (2) The structure of DLMNP loaded with dual drugs. (3) Schematic of the preparation method of ammonia borane-loaded mesoporous silica nanoparticles (AB-MSN) and the mechanism of acid-triggered decomposition and H_2_ release. (4) Schematic of smart thermal-responsive drug release of the SMND controlled by a smartphone. (5) Schematic illustration of proposed mechanism using SMND for anti-CSC synergistic immunity/hydrogen therapy. Reprinted with permission from [149]. Copyright 2022 John Wiley & Sons.

It is usually difficult to achieve the best therapeutic effect by a single antitumor therapy, and a more effective treatment can often be achieved by adopting a synergistic therapy. Surgical removal of superficial tumors and surrounding skin tissue is the mainstay of treatment for malignant skin tumors, but the risk of postoperative residual infiltrating tumor cells and wound infection still remains [131]. Lei et al. [132] introduced SiO_4_^4−^ onto the surface of natural melanin NPs extracted from cuttlefish ink (CINP) by biomimetic mineralization and prepared CINP@SiO_2_ NPs with good bioactivity, which then combined with MeHA MN patches to achieve the dual efficacy of tumor PTT and wound healing (Fig. 9A). 3-(4,5-Dimethylthiazol-2-yl)-2,5-diphenyltetrazolium bromide (MTT) experiments showed that CINP@SiO_2_ had no obvious cytotoxicity after coculture with L929 mouse fibroblast cells for 24 h. The cell proliferation rate of CINP@SiO_2_-HA MNs cocultured with HUVECs for 3 d was determined by cell counting kit-8 (CCK-8) method. The migration experiments of HUVECs showed that the CINP@SiO_2_-HA MN group had the highest mobility and could promote the migration of HUVECs. The expressions of hypoxia-inducible factor 1α (HIF-1α), VEGF, kinase insertion domain receptor (KDR), and endothelial NO (eNOs) were also measured by enzyme-linked immunosorbent assay (ELISA), and it was found that the CINP@SiO_2_-HA MN group had the highest expression effect, indicating that CINP@SiO_2_-HA MNs had the ability to promote angiogenesis. In vitro antitumor experiments showed that the CINP@SiO_2_-HA MN group had the best photothermal treatment effect. In vivo animal experiments confirmed that CINP@SiO_2_-HA MNs could simultaneously achieve antitumor and skin tissue regeneration effects through PTT, ROS scavenging, and up-regulation of angiogenic gene expression. Accordingly, the CINP@SiO_2_-HA MN patch has valuable application potential.

Because traditional chemotherapy is a systemic treatment method, where the selectivity of chemotherapy drugs is not strong, it inevitably damages normal cells while killing tumor cells, resulting in large adverse reactions [114]. The MN patch can achieve local and efficient delivery of drugs, and further combination of PTT and chemotherapy with MN patch can maximize the tumor cell killing efficiency and effectively reduce the occurrence of adverse side effects. Moreira et al. [133] used electrospraying process to spray doxorubicin (DOX)-loaded chitosan and gold-core mesoporous silica shell (AuMSS) nanorod-rich PVA layer by layer on the surface of PVP MN substrate to prepare DOX@MicroN patches. The cytotoxicity of the DOX@MicroN patch on HeLa cancer cells was evaluated in vitro. With the combined effect of PTT and Dox, the cell viability of HeLa cancer cells was reduced to 3.8%, with extremely high cytotoxicity to achieve prominent tumor cell killing effect. Loading gold nanocages (AuNCs) and DOX into soluble HA MNs can enhance the strength of MNs to effectively pierce into skin and subsequently release AuNCs and DOX to the tumor site, with a synergistic and effective antitumor effect of chemotherapy and PTT [134]. Paclitaxel and indocyanine green (ICG)-loaded α-tocopherol polyethylene glycol succinic acid (TPGS)/HA bifunctional PLGA NPs can be used as chemotherapeutic drugs and photothermal agents and then encapsulated into PVP/PVA to prepare soluble MN patches with chemotherapeutic and PTT functions, which can effectively inhibit the proliferation of 4T1 tumors in mice [135]. Loading multifunctional materials with properties such as PTT [136], PDT [137], immunotherapy [138], chemodynamic therapy (CDT) [139], and other properties [140] into MN patches for synergistic therapy can significantly improve the therapeutic effect of tumors and has broad application prospects (Fig. 9B and C) [141–145].

**Fig. 10.**
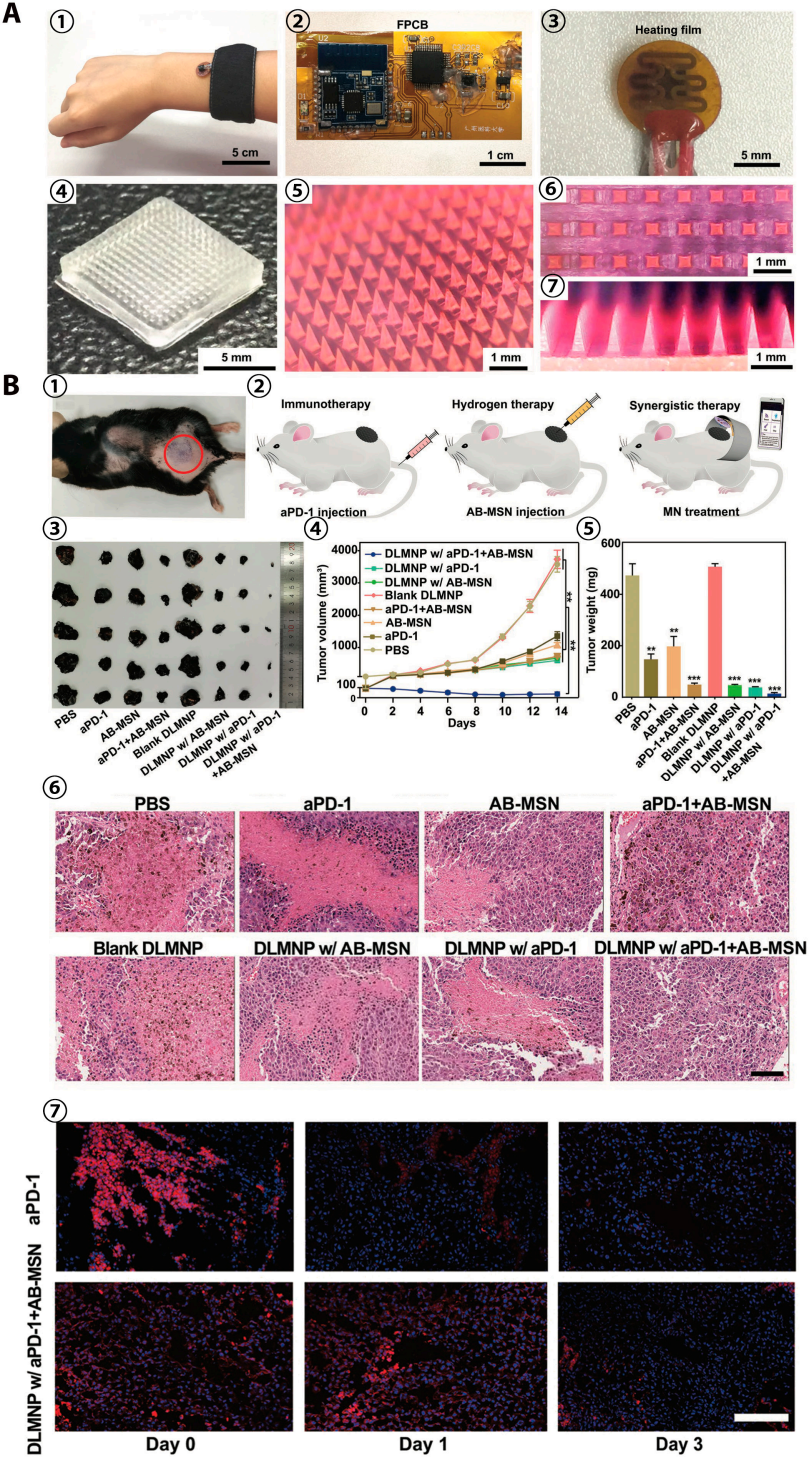
SMND for the synergistic antitumor effect of immunotherapy and hydrogen therapy. (A) Electronic and physical structure of an SMND. (1) Optical image of the assembly of SMND with an FPCB under the wrist strap. (2) Optical image of detailed block circuit on FPCB. (3) Optical image of flexible heating film that was electrically connected with FPCB. (4) Digital photograph of a DLMNP. (5) Digital microscope image of DLMNP dyed with rhodamine 6G and its platform (6) as well as side view (7). (B) In vivo synergetic anticancer efficiency of SMND. (1) Mouse dorsum and relevant skin (the area within the red circle) was transcutaneously treated with a DLMNP of SMND. (2) Schematic of the different treatments performed on the B16F10-CSC tumor-bearing mouse model. (3) Representative photographs of tumors from treated mice. (4) Tumor growth curves and (5) tumor weight of B16-CSC tumor-bearing mice within 14 d of treatment. (6) Representative H&E-stained images of excised tumors after 14 d of treatment. Scale bar is 100 μm. (7) Immunofluorescence staining of tumors at different time points (green: aPD-1, blue: nucleus, scale bar is 100 μm). Reprinted with permission from [149]. Copyright 2022 John Wiley & Sons.

Melanoma cancer stem cells (CSCs) are one of the constituents of melanoma, which can lead to melanoma recurrence and ineffective treatment [146]. The aPD-1 immunotherapy can effectively treat CSC, but the evolution of immune evasion in CSC will result in gradually limited antitumor effect [147]. Hydrogen has anti-inflammatory and antitumor effects, and hydrogen therapy is a potential antitumor strategy [148]. MNs can deliver drugs into subcutaneous tumor tissue to achieve high-efficiency drug delivery, so the combination of immunotherapy, hydrogen therapy, and MNs can maximize the anti-CSC and antitumor effects. Yang et al. [149] developed a wearable silk-based microneedle device (SMND) for the synergistic antitumor effect of immunotherapy and hydrogen therapy (Fig. 9D). SMND can achieve thermally responsive hydrogen release through temperature control via mobile phone Bluetooth and can also continuously release aPD-1 in tumor tissue to achieve sustained immunotherapy effects. This synergistic treatment combining hydrogen therapy and immunotherapy of SMND achieved the best anti-CSC and antitumor effects in mouse in vivo experiments and is a potential melanoma treatment strategy (Fig. 10).

#### Treatment of diabetes

Insulin injections are the most common treatments for diabetes, but the pain and discomfort caused by intravenous injections induce the physical and mental inconvenience for diabetic patients [150]. MNT can achieve painless drug delivery, and encapsulating insulin into MN to achieve its effective delivery can play a role in the treatment of diabetes [151]. Long-acting insulin delivery is more effective for diabetes treatment, so Chen et al. [152] designed an MN patch that can deliver insulin sustained in long term to achieve a long-acting glycemic control. Two types of insulin long-release MN patches were prepared by combining MN matrix materials with different degradation rates or encapsulating insulin with different action time. The results of in vivo experiments in diabetic rats showed that only one insertion of the MN patch could achieve sustained release of insulin within 1 d, which had a prominent therapeutic effect on diabetes.

**Fig. 11.**
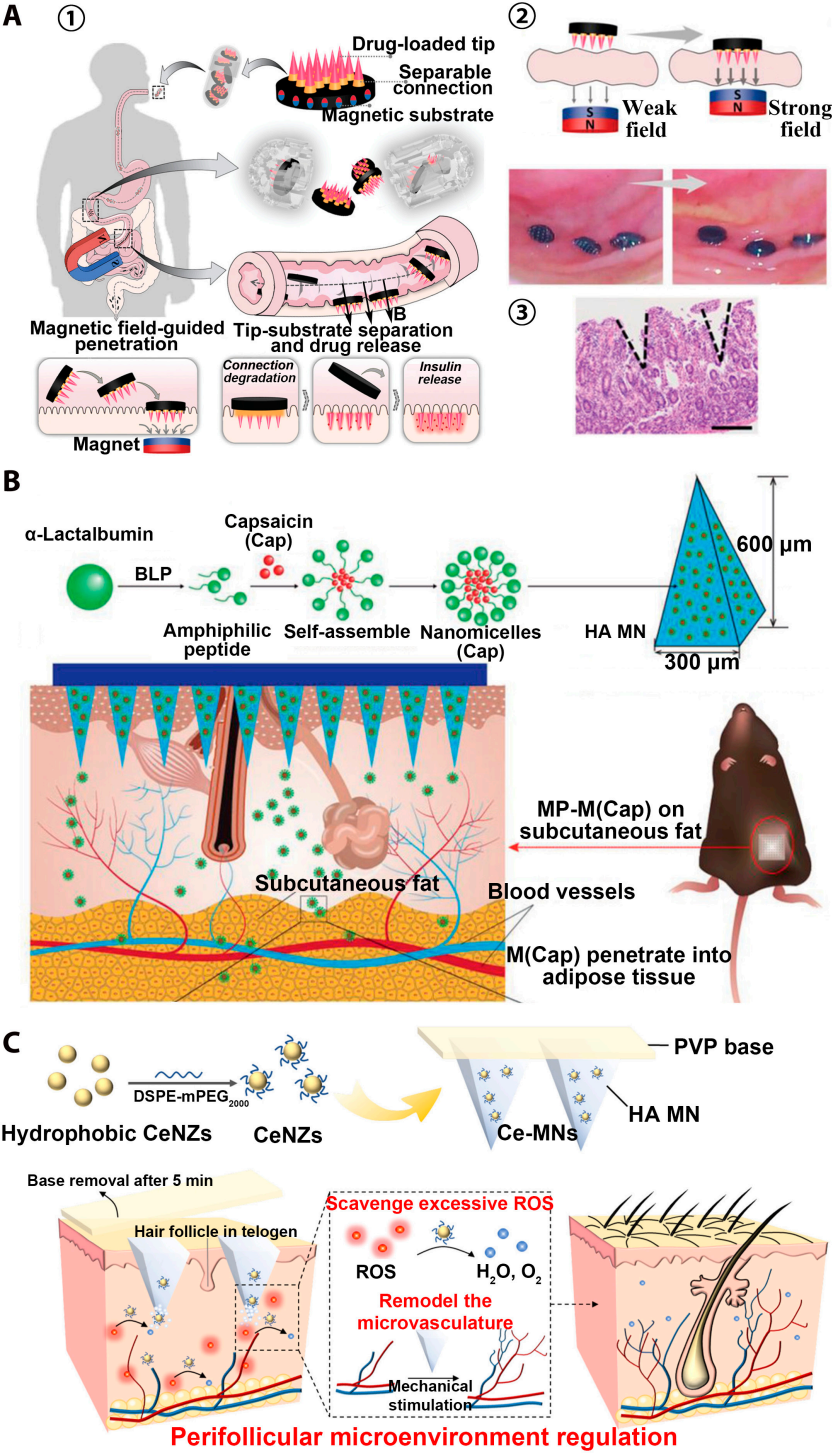
MNT for diabetes, obesity, and alopecia treatment. (A) Magnetic MN enables efficient delivery of insulin in the gut for diabetes. (1) Schematic diagram of magnetic-responsive MN robot therapy. After being swallowed into the small intestine, MN robot penetrated into the small intestine under the action of a magnetic field to release insulin. (2) Scheme and digital images of MN robots penetrating the small intestine tissue after magnetic field enhancement. (3) H&E staining of the MN robot-penetrated small intestine. Reprinted with permission from [154]. Copyright 2021 John Wiley & Sons. (B) HA MN patch delivers Cap encapsulated in α-lac nanomicelles to adipose tissue for obesity treatment. M(Cap) self-assembled from α-lac peptide and Cap were loaded into HA MNs, and M(Cap) promoted white fat browning and inhibited lipogenesis. Reprinted with permission from [158]. Copyright 2021 John Wiley & Sons. (C) MN patches loaded with ceria nanozymes (CeNZs) achieve alopecia efficacy through mechanical stimulation and scavenging of excess reactive oxygen (ROS) species. CeNZs could be directly transported to the dermis and epidermis through MNs to remove excess ROS, and the microvascular system in the microenvironment around the HFs could be remodeled by MN-induced mechanical stimulation to promote angiogenesis and transform the HFs from the resting phase to the anagen phase. Reprinted with permission from [167]. Copyright 2021 American Chemical Society.

Oral administration is the most convenient and common way of administration, but the difficulty in overcoming the complex gastrointestinal barrier and in achieving efficiently targeted absorption of macromolecular drugs still remains a challenge [153]. Using a multilevel 3D fabrication strategy inspired by stacking Lego bricks, Zhang et al. [154] developed a magnetically responsive MN robot enabling macromolecular drug delivery (Fig. 11A) [154]. The magnetic substrate, separable connection, and tip made up the MN robot. The MN robot was encapsulated in a commercial enteric-coated capsule that allowed it to pass through gastric fluid and be released in the small intestine. Under the magnetic field, the MN robot could overcome the small intestinal barrier and insert into the tissue, and the degradation of the detachable junction allowed the tip to remain in the small intestinal tissue while the magnetic substrate was excluded from the body. Insulin was loaded into an MN robot for oral delivery to pigs to achieve effective blood glucose control. These results suggest that the MN robot is an effective platform for the oral drug delivery of macromolecules. Caffarel-Salvador et al. [155] chose a different strategy to achieve efficient absorption of macromolecular drugs. Using the oral buccal mucosa as the action site of MN patch, a 1-mg payload of human insulin was delivered to the oral cavity of pigs within 30 s, achieving faster macromolecular drug delivery than on the skin.

#### Treatment of obesity

Obesity is a serious problem facing mankind all over the world, which trigger a variety of metabolic disorders and numerous diseases such as hyperglycemia and blood pressure increase, insulin resistance, type 2 diabetes, cancer, and cardiovascular and cerebrovascular diseases [156]. Extensive efforts have been focused on treating obesity, with methods including dietary calorie restriction, physical activity, administration of anti-lipogenic drugs, gastric surgery, and liposuction, whereas more aggressive anti-obesity treatments can cause serious adverse side effects [157]. Highly effective obesity treatments using minimally invasive and adipocyte-specific anti-obesity techniques would be more attractive. As a painless, minimally invasive, and efficient treatment method, MNT will definitely have broad application potentials in the field of obesity treatment. Bao et al. [158] prepared M(Cap) formulations by encapsulating capsaicin (Cap) with anti-obesity effect in α-lactalbumin (α-lac) nanomicelles through self-assembly method and combining HA and an MN patch with body temperature-responsive melting properties [MN-M(CAP)] made of PVA to deliver M(Cap) directly to adipose tissue for obesity treatment (Fig. 11B). Through the treatment of high fat diet (HFD)-induced obese mice, MN-M(CAP) achieved a rather significant obesity therapeutic effect compared with direct subcutaneous injection of M(Cap) formulation, indicating that bioavailability of Cap could be greatly enhanced through α-lac encapsulation. β3-Adrenergic agonists (CL316,243) are anti-obesity drugs with apparent efficacy, but certain side effects to the body could be caused by traditional methods of administration such as oral and intravenous injections [159]. Than et al. [160] combined 2 anti-obesity compounds, CL316,243 and thyroid hormone T3, with the HA MN patch for direct drug-targeted subcutaneous white adipose tissue (WAT) delivery. The results of in vivo animal experiments showed that the adipose tissue weight of mice treated with CL316,243-MN patch was significantly reduced, while the adipose tissue weight of mice injected with the same dose of CL316,243 by intraperitoneal injection was almost unchanged. The potential of MN patches with obesity treatment effect is significant. Rosiglitazone (Rosi) is a thiazolidinedione drug used in the treatment of type 2 diabetes and also has a role in the treatment of obesity [161]. Rosi was loaded into the MeHA MN patch, and BP was coated on the surface of the MN backing, which effectively promoted the release of Rosi in vivo through photothermal regulation of temperature, achieving an effective treatment for obese model mice [162].

#### Treatment of alopecia

Vascular insufficiency and/or oxidative stress in peri-follicular microenvironment can cause androgenetic alopecia (AGA) [163]. Delivering anti-hair loss drugs through MNT can present good anti-hair loss effect. Valproic acid (VPA), a Food and Drug Administration (FDA)-approved anticonvulsant drug, is more potent than minoxidil in inducing HF regeneration [164]. Dissolvable MN patches loaded with VPA were prepared by CL [165]. Compared with the direct application of VPA, the MN patch not only significantly improved the delivery efficiency of VPA but also promoted the differentiation of HF stem cells through the mechanical stimulation of MN, achieving prominent effect on boosting HF regeneration. Cerium dioxide nanozymes (CeNZs) can scavenge excess ROS and reduce oxidative stress [166]. Therefore, CeNZs and MN patches could be combined to design Ce-MN patches, which take advantage of the mechanical strength of MN to form micropores on the skin to facilitate the delivery of CeNZs (Fig. 11C) [167]. Compared with FDA-approved alopecia treatment drug minoxidil, Ce-MNs achieve faster regeneration of the same quality of hair with lower administration frequency and no irreversible damage to the skin, which has great potential for the treatment of AGA. Kim et al. [168] combined minoxidil with HA to prepare a dissolvable HA-MN patch for AGA treatment. Cell adhesion and cell function can be promoted by HA to help hair growth, minoxidil and HA synergistic treatment can significantly promote hair growth, and HA-MN has the least side effects and also shows the best AGA treatment effect. Finasteride (FIN), another FDA-approved AGA treatment, inhibits HF atrophy by reversing the process of AGA by inhibiting the 5α-reductase type 2 (SRD5A2) gene in scalp HF [169]. FIN requires long-term oral administration to achieve a better therapeutic effect, but long-term oral FIN may cause serious adverse reactions. Lipid nanocarriers have good affinity for HF and can be used as delivery vehicles for FIN. MN can overcome the SC barrier to achieve efficient deep delivery of drugs. The delivery of FIN-loaded lipid nanocarriers (FIN-NLC) through MN into HF can effectively up-regulate hair growth-promoting signals and down-regulate inhibitory signals, achieving a highly prominent therapeutic effect of AGA [170]. Combining FIN with soluble PVP/PVA MN or degradable PLGA MN can also achieve long-lasting FIN release up to 7 and 14 d, respectively [171].

In addition to traditional drugs, some natural active ingredients combined with MN can also effectively treat AGA. β-Sitosterol is a natural phytosterol that exists in multiple parts of plants and has various pharmacological activities such as angiogenesis, antioxidant, and immunomodulation [172]. Encapsulating β-sitosterol in lipid nanocarriers and achieving sustained deep and long-lasting delivery of β-sitosterol through degradable MN achieved an effective treatment for AGA [173]. Alopecia areata (AA) is a complex immune-mediated hair loss disease, and it is difficult to achieve effective treatment due to its complex etiology [174]. MN can induce tissue regeneration by promoting the release of growth factors through minimally invasive mechanical stimulation. Giorgio et al. [175] applied the PDT photosensitizer 5-aminoacetic acid (ALA) to the scalp and subsequently inserted metal MNs under the skin to achieve synergistic treatment of the moderate to severe AA using PDT and MN. MNs can promote the absorption of ALA to achieve better PDT effect, and the combined treatment of PDT and MN has the optimal therapeutic effect on the moderate to severe AA.

#### Treating skin, heart, and other diseases

**Fig. 12.**
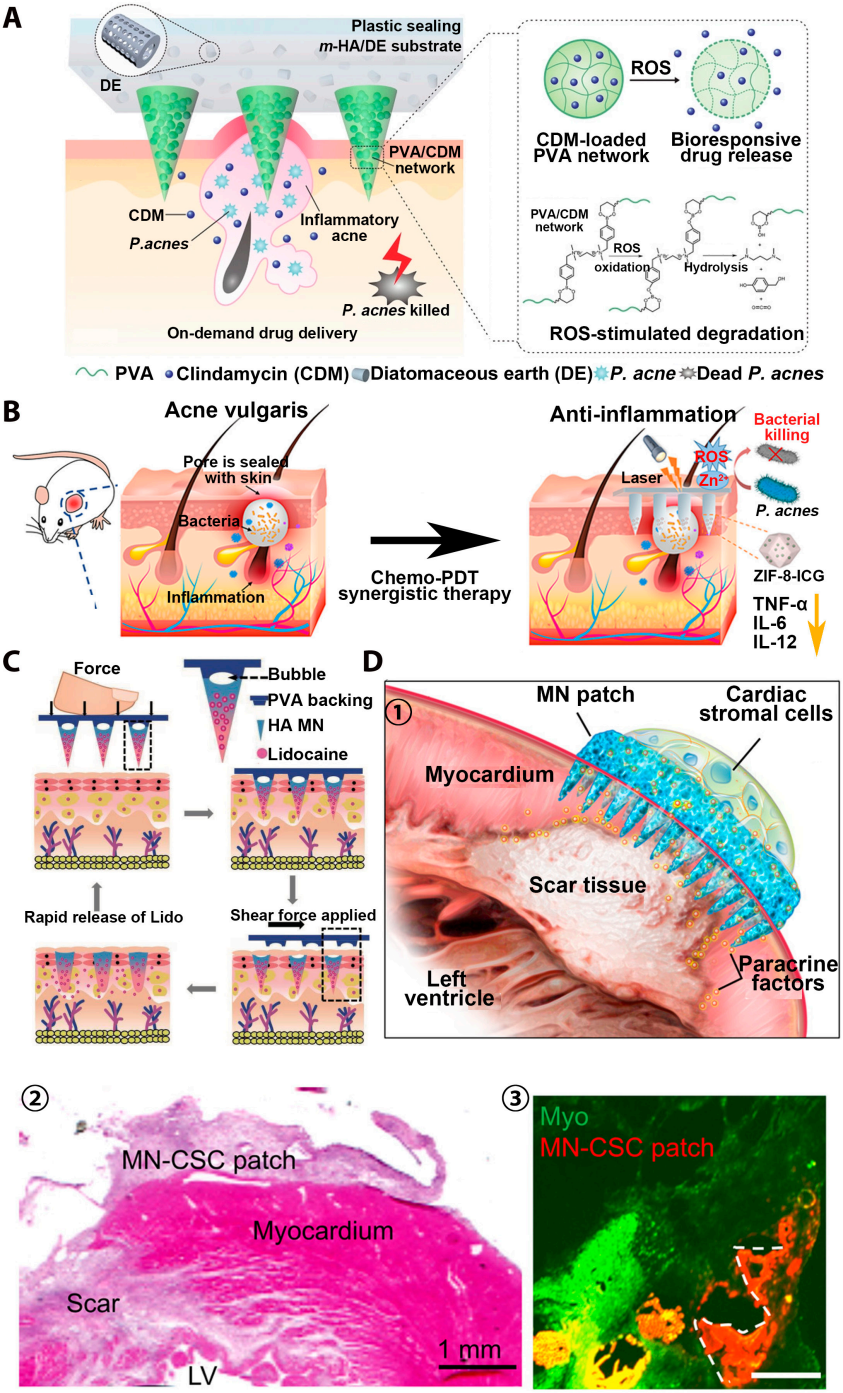
MNT for other disease treatment. (A) Antibiotic-delivered MN patch for the treatment of acne vulgaris. ROS-responsive MNs could sustainably release drugs in *P. acnes*-infected HFs after penetrating the epidermis to effectively inhibit bacterial proliferation. Reprinted with permission from [178]. Copyright 2018 John Wiley & Sons. (B) MN patch with chemo-photodynamic therapy for the treatment of acne vulgaris. The ICG photosensitizer was modified into ZIF-8 to generate ROS by PDT, and ZIF-8 was degraded under acidic conditions to sustain release of Zn^2+^ to kill *P. acne*. Reprinted with permission from [179]. Copyright 2021 American Chemical Society. (C) High-dose analgesic drug LiH-loaded MN patch for local durable analgesia. The air bubble structure between the MNs and backing layer enabled the MNs to effectively penetrate into the skin and be removed from the backing layer under the action of shear force, thereby completing drug delivery quickly. Reprinted with permission from [182]. Copyright 2022 Springer Nature. (D) Cardiac stromal cell-loaded MN (MN-CSC) patch for myocardial infarction therapy. (1) Schematic diagram of MN-CSC treatment of myocardial infarction. Infarcted heart could be effectively treated through vascular myogenesis, reduction in scar size, and enhancement of cardiac function by MN-CSC patch. (2) H&E staining after MN-CSC patch applied on the infarcted heart. Scale bar is 1 mm. (3) Fluorescent image of Cy5.5-labeled MNs (red) on the heart (green) 7 d after the transplantation. Scale bar is 400 μm. Reprinted with permission from [185]. Copyright 2018 American Association for the Advancement of Science.

Encapsulating drugs for different diseases into MNs can achieve efficient transdermal delivery of drugs, so MNs have therapeutic effects on various diseases. Acne vulgaris is a common chronic inflammatory skin disease, and excessive colonization of *Propionibacterium acnes* is one of the causes of acne [176]. The traditional treatment method is to apply anti-acne cream on the skin surface, but due to the barrier of skin SC, most active ingredients are difficult to penetrate into the subcutaneous lesions, so the therapeutic effect is limited [177]. Loading drugs into the MN patch can effectively break through the keratinous barrier, enabling drug delivery into the dermis to achieve excellent acne treatment effects (Fig. 12A) [178]. The MN patch prepared by loading a photosensitizer ICG into MOF and combining with HA can effectively kill *P. acnes* by PDT to achieve excellent anti-acne effect (Fig. 12B) [179]. In addition to the treatment of acne, the combination of PDT and MN can also successfully treat skin diseases such as hypertrophic scars and cutaneous warts, indicating that MNT possesses high potential for applications in treatment of skin diseases [180,181]. Local skin analgesia can also be achieved with MNT [182]. Loading high-dose analgesic drug lidocaine hydrochloride (LiH) into the GelMA MN patch can significantly enhance and prolong the anesthetic effect of LiH to achieve local durable analgesia in rats (Fig. 12C) [183]. Encapsulation of the MSCs into GelMA MNs with a PLGA shell also enables the efficient delivery of MSC for regenerative therapy [184]. In addition to the skin treatment, significant application of cardiac stromal cell-loaded MN patches to the heart can effectively promote cardiac function and facilitate cardiac regeneration for the treatment of myocardial infarction (Fig. 12D) [185].

### Medical diagnosis and health monitoring

#### Extraction of skin ISF

**Fig. 13.**
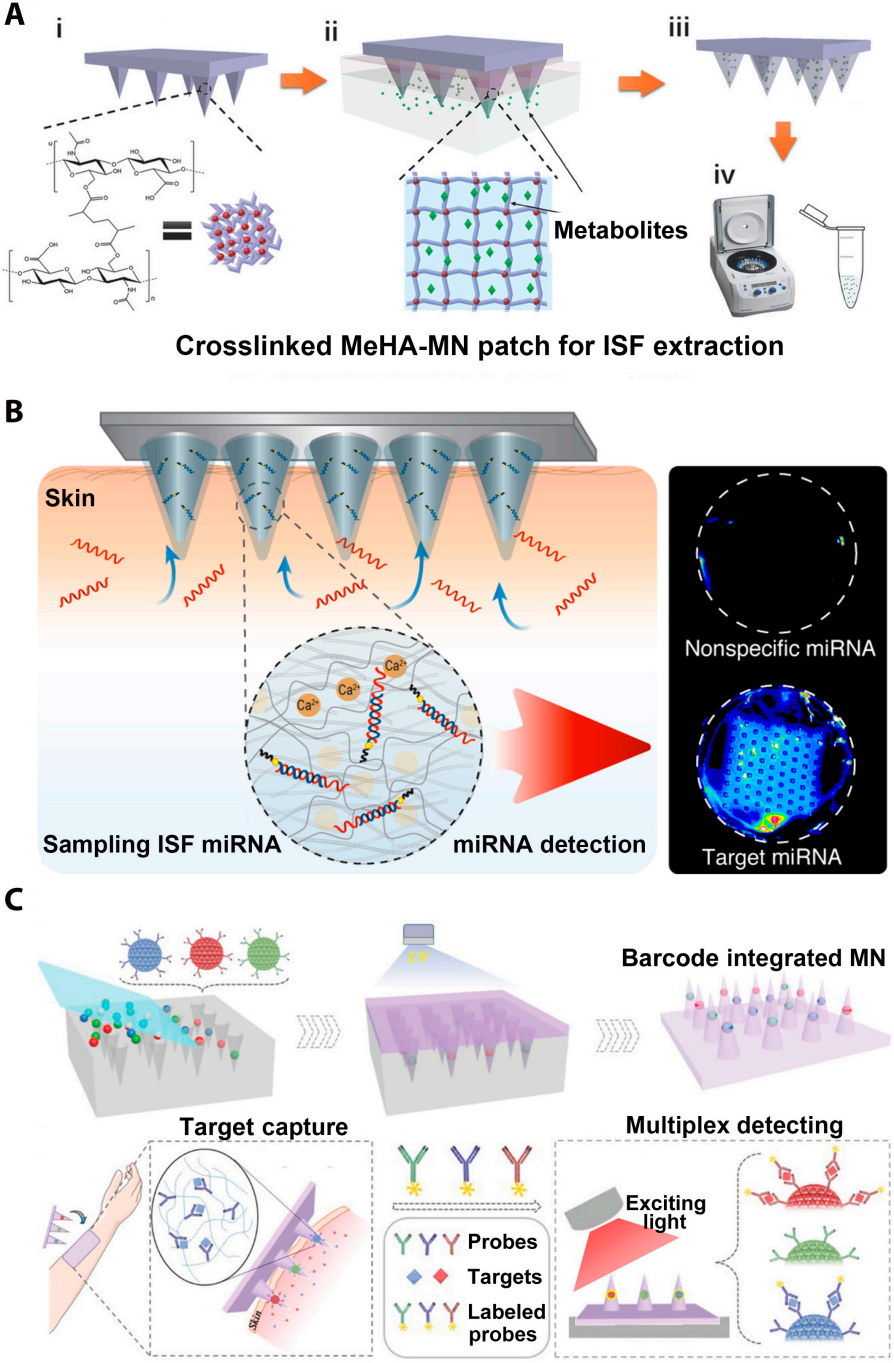
MNT for extraction and utilization of ISF. (A) MeHA hydrogel MN patch for skin ISF extraction. Skin ISF could be rapidly extracted by the swelling of MeHA MN patch and be recovered from MN patch by centrifugation. Reprinted with permission from [187]. Copyright 2017 John Wiley & Sons. (B) Alginate–PNA hydrogel-coated PLLA MN for miRNA detection in ISF. PNA probes in alginate coating capture could target miRNA in ISF for detection. Reprinted with permission from [189]. Copyright 2019 American Chemical Society © 2019. (C) Integrating high-throughput monitoring technology PhC barcodes into MN for biomarker analysis in ISF. The relative content and specific species of biomarkers in ISF could be analyzed by PhC barcode intensity and different reflection peaks, respectively. Reprinted with permission from [191]. Copyright 2019 John Wiley & Sons.

**Fig. 14.**
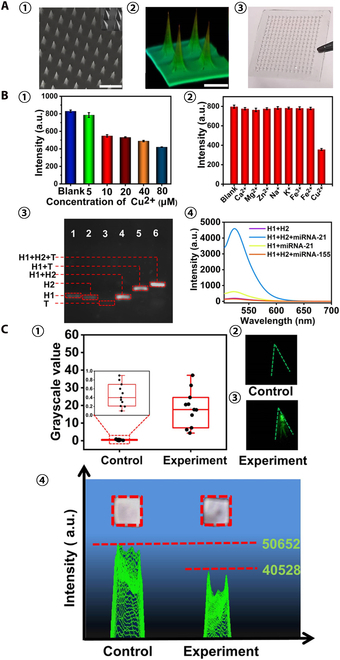
Cross-linked c-GelMA-MeHA MN patch for ISF extraction. (A) (1) SEM image, (2) 3D fluorescence image, and (3) optical image of MN patch. Scale bars are 2 mm in (1) and 1 mm in (2). (B) (1) Fluorescence spectra of carbon quantum dots (CQDs) with different concentrations of Cu^2+^ ions. (2) Fluorescence spectra of CQDs with different metal ions. (3) Feasibility analysis for miRNA-21 detection. (4) Feasibility analysis for miRNA-21 detection. (C) (1) Fluorescence intensity of the corresponding MNs with different concentrations of miRNA-21 and (2) the representative fluorescence images. Scale bar is 100 μm. (3) Fluorescence intensity of the corresponding MNs with different concentrations of Cu^2+^ and (4) the representative grayscale images. Reprinted with permission from [190]. Copyright 2022 American Chemical Society.

Skin ISF is formed by blood transcapillary exchange, similar to plasma composition, and contains a large number of biomarkers whose contents vary with physiological changes; therefore, ISF has great potential in minimally invasive diagnostics and sensors [186]. The hydrogel MN patch has excellent swelling ability to achieve efficient ISF collection. The MeHA synthesized by modifying HA with methacrylic anhydride can be cross-linked by radical polymerization under UV irradiation, maintaining the structural integrity of HA while retaining good mechanical properties. The MeHA MN patch has remarkable hydrophilic and swelling ability, which can extract ISF from mouse skin within 10 min, which has a promising application prospect (Fig. 13A) [187]. Taking advantage of ultrahigh biocompatibility, gelatin methacryloyl (GelMA) is an excellent choice for preparation of ISF MN patches used for minimally invasive extraction. The swelling ratio and mechanical properties of the patches could be optimized by adjusting the concentration and cross-linking time of GelMA prepolymer to achieve efficient and rapid extraction of ISF [188]. Al Sulaiman et al. [189] coated alginate–peptide nucleic acid (PNA) hydrogel on the surface of PLLA to prepare an MN patch that could rapidly extract and detect nucleic acid biomarkers in ISF (Fig. 13B). Up to 6.5 μl of ISF could be collected by MN within 2 min, and specific miRNA biomarkers could be isolated from it for in situ detection. Li et al. [190] used GelMA and MeHA as MN matrix materials and encapsulated the miRNA and Cu^2+^ detection systems, realizing the minimally invasive and efficient detection of miRNA and Cu^2+^ in ISF, which has promising application prospects (Fig. 14). Integrating photonic crystal (PhC) barcoding, a high-throughput monitoring technology, into MNs can realize the effective analysis of the type and relative content of biomarkers in ISF, with the advantages of high efficiency and versatility, which is of great significance for disease monitoring and screening (Fig. 13C) [191].

#### Tumor diagnosis

**Fig. 15.**
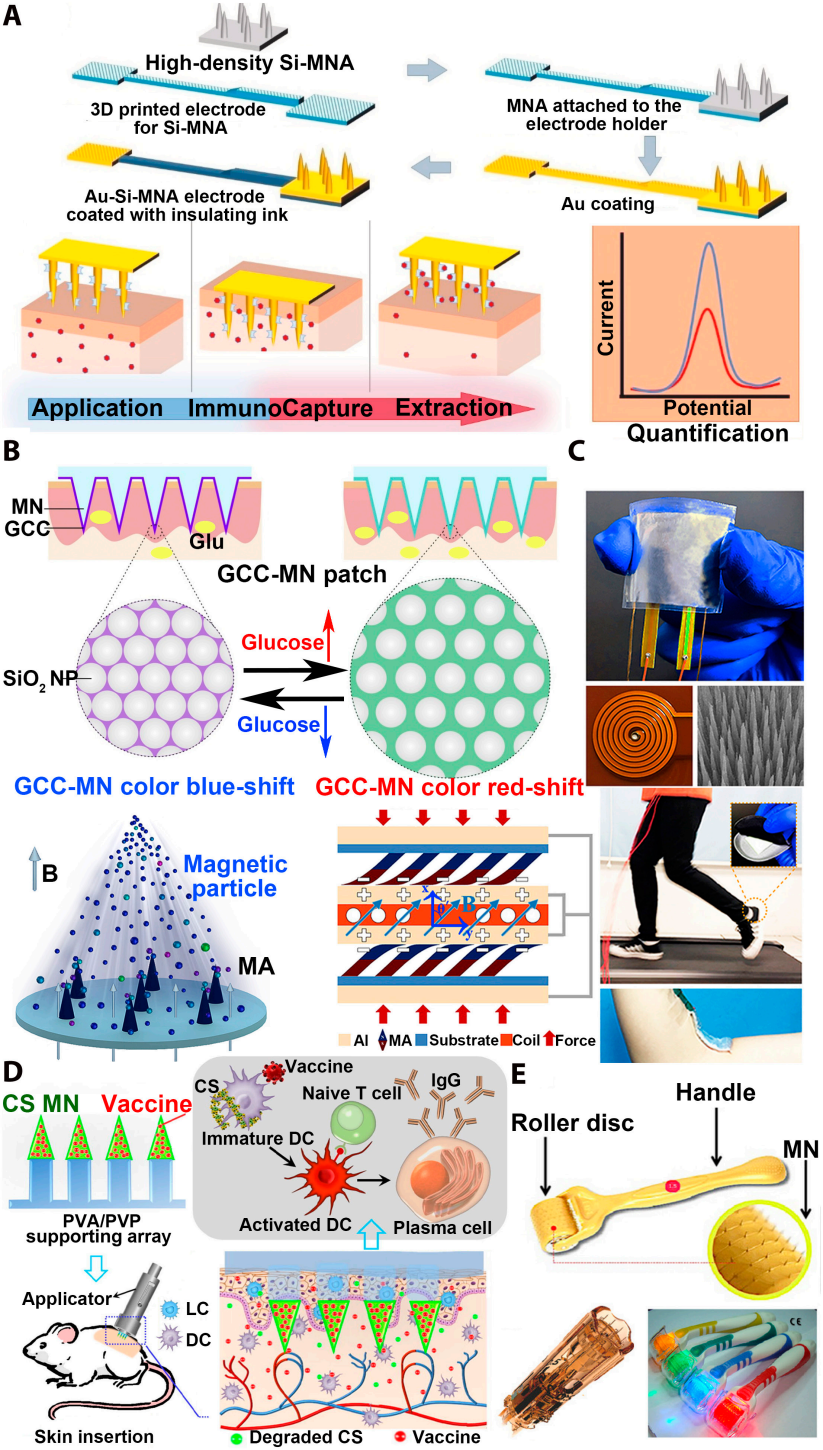
MNT for medical diagnosis, health monitoring, vaccination, and medical beauty. (A) Silicon MN electrochemical sensor quantifies breast cancer biomarker ErbB2 for tumor detection. Anti-HER2 antibody was bound to 3-mercaptopropionic acid-functionalized Au-Si-MNA electrode to detect ErbB2 in artificial ISF. Reprinted with permission from [193]. Copyright 2021 Elsevier. (B) GCC color change on the surface of MN realizes blood glucose monitoring with naked eye recognition. The GCC-MN patch could convert glucose concentration into a color change that could be discerned by the naked eye within 5 min. Reprinted with permission from [196]. Copyright 2020 Elsevier. (C) TEHG based on magnetic MAs for motion monitoring. The MA-based TEHG was assembled in the insole as a wearable pedometer to accurately track walking/jogging strides and mounted on the inside of the elbow as a bendable sensor that also correctly monitors the rotation of the arm. Reprinted with permission from [199]. Copyright 2020 Elsevier. (D) Chitosan (CS) MNs are used for influenza vaccination. CS MN acted as an antigen depot and immunizing agent for sustained release of vaccine and immune activation after easy skin implantation. Reprinted with permission from [203]. Copyright 2019 Elsevier. (E) Mechanical stimulation of solid MNs induces skin elastin and collagen expression and deposition for anti-aging. Solid MNs have been widely used in the field of medical beauty. Reprinted with permission from [207]. Copyright 2015 John Wiley & Sons.

Breast cancer is among the 5 most common causes for cancer death, and monitoring breast cancer biomarkers is critically important for early diagnosis of breast cancer [192]. Epidermal growth factor receptor 2 (ErbB2) is an important breast cancer biomarker, and both the extraction and quantification of ErbB2 can be achieved by the silicon MN electrochemical sensor simultaneously, which is of great significance for the prevention and early detection of breast cancer (Fig. 15A) [193]. The silicon MN sensing platform integrates functionalized nano-gold-coated silicon MNs into a 3D-printed scaffold, and subsequently, the MNs are inserted into skin to quantify the captured biomarkers for a simple and fast diagnosis.

#### Blood glucose monitoring

The traditional blood glucose measurement requires a lancet to draw blood from a finger, which not only causes pain and discomfort but also makes it difficult to continuously measure blood glucose [194]. ISF can be extracted by a minimally invasive MNT method to replace blood for blood glucose monitoring, which is more convenient for continuous monitoring. Dervisevic et al. [195] fabricated microelectrodes by combining high-density silicon MN patches with 3D-printed scaffolds. The surfaces of the MNs were coated with different materials to form a 3-electrode system including the working electrode, reference electrode, and counter electrode. In situ minimally invasive extraction of ISF and continuous blood glucose monitoring could be achieved simultaneously, and the measured blood glucose levels had good correlation with the commercial blood glucose meters. Zeng et al. [196] designed an MN patch containing glucose-responsive colloidal crystals (GCCs), combining soft GCCs with hard transparent resins to prepare core-shell structured MN patches (Fig. 15B). With the gradual increase of the glucose concentration, the MNs were altered from green to yellow to red, and the blood glucose monitoring could be easily realized by the naked eye through the color change.

#### Motion monitoring

Wearable electronics can allow people to perceive changes and multiple information of their bodies more intuitively [197]. Triboelectric–electromagnetic hybrid generators (TEHGs) can convert biomechanical energy generated by the human body into electrical energy [198]. Based on magnetized MAs, Li et al. [199] developed a novel flexible TEHG for monitoring human motion (Fig. 15C). A mixture of polydimethylsiloxane (PDMS) and toluene containing magnetic NPs and 10% curing agent was used as the prepolymer solution, and the prepolymer solution was sprayed from a spray gun to form an aerosol that was sprayed onto the PDMS substrate, followed by the formation of magnetized MA through magnetic field-induced spray self-assembly, thermal curing, and electromagnetization. The MA-based TEHG can be used both as a wearable pedometer to discipline steps and as a bendable sensor to enable monitoring of arm rotation.

### Transcutaneous immunization

**Fig. 16.**
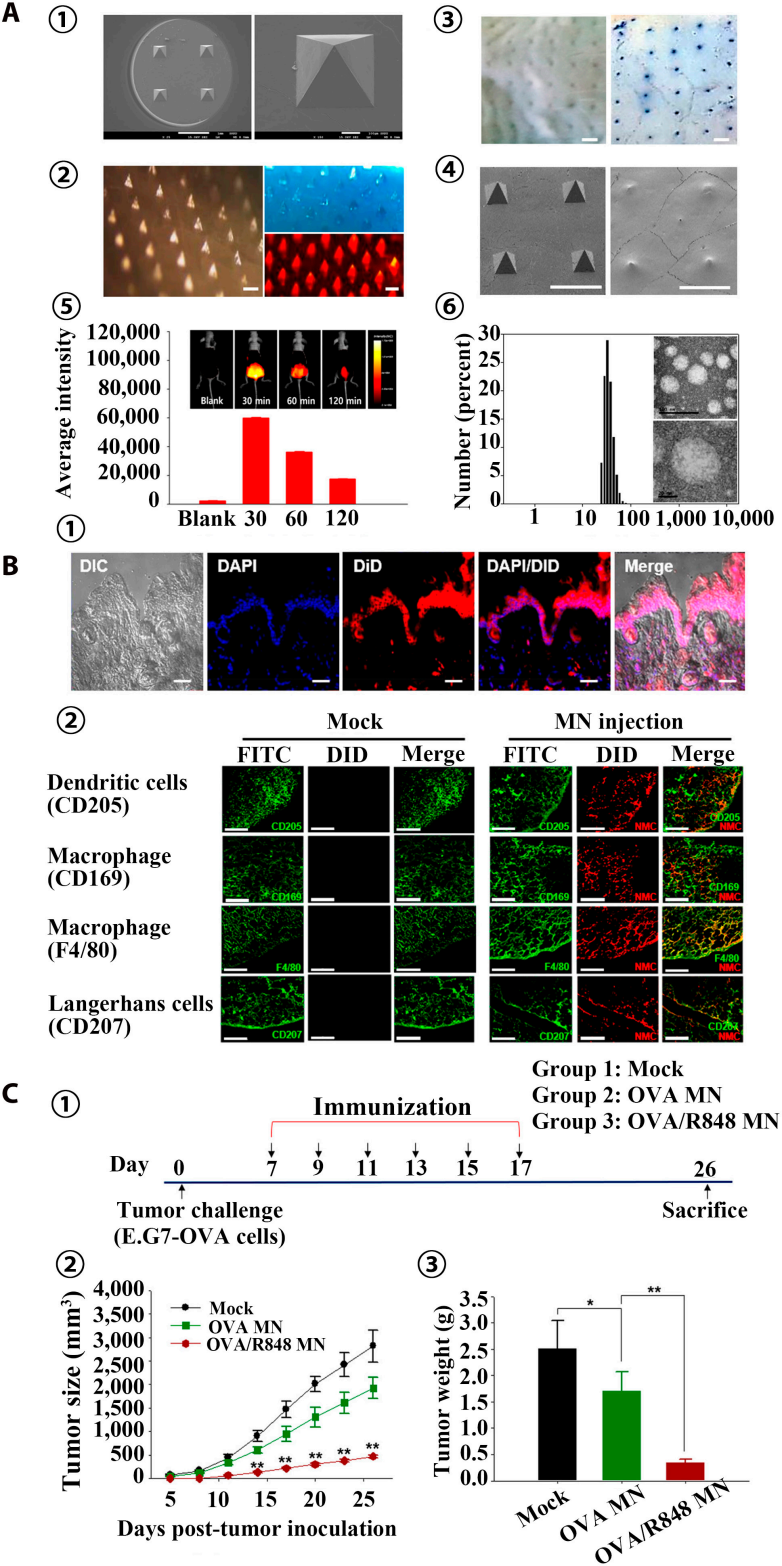
Soluble MNs for TCI. (A) Characterization of NMC-generating dissolving MNs. (1) SEM images. Scale bars are 1 mm and 100 μm (25×, 150×). (2) Fluorescence stereomicroscope images of DiD-loaded MNs. Scale bars are 500 μm. (3) Penetration of MNs into the back skin of a mouse, which was stained with trypan blue solution. Scale bars are 1 mm. (4) SEM images of MNs before and after application to mouse skin. Scale bars are 1 mm. (5) In vivo distribution of DiD-loaded NMCs from MNs after injection. (6) Size distribution and transmission electron microscopy images of NMCs generated by the dissolution of MNs in PBS. (B) (1) Intradermal delivery of DiD@NMCs generated from MNs after injection into mouse skin. Cell nuclei (blue) were dyed with 4′,6-diamidino-2-phenylindole (DAPI). Scale bar is 50 μm. (2) In vivo trafficking of DiD@NMCs released from MNs to lymph nodes. Scale bars are 90 μm. (C) (1) Immunization schedule for therapeutic vaccination for an established solid tumor. (2) Therapeutic antitumor immunity of OVA/R848 MN. The mock group was injected with PBS. (3) Average tumor weights from the 3 experimental groups. Reprinted with permission from [205]. Copyright 2018 American Chemical Society.

Vaccines are usually administered intramuscularly to elicit an immune response, but TCI can also elicit an immune response comparable to or higher than intramuscular injection [200]. Combining vaccines or synthetic nanovaccines with MNs can achieve painless subcutaneous drug delivery to achieve preventive effects on the corresponding diseases. Choi et al. [201] encapsulated canine influenza vaccine into MN tips composed of soluble HA and employed PCL as the backing layer of the MN to prepare a tip-detachable MN patch. The soluble tips can detach immediately after MN is inserted into the skin, releasing the vaccine components in the tip coating to elicit an immune response. Through in vivo experiments on guinea pigs, it was found that the level of specific antibodies in the serum after MN insertion was significantly increased, indicating that the vaccination has been successfully achieved. Chitosan is a commonly used vaccine adjuvant with excellent biocompatibility and biodegradability [202]. The MN patches prepared by taking chitosan as the MN tips and encapsulating the influenza vaccine in it, and using PVA/PVP as the MN backing layer, can release the vaccine through the slow degradation of chitosan into skin and achieve long-term immune stimulation under the cooperative effect of chitosan, inducing the continuous production of specific antibodies, with successful achievement on remarkable immune protection effect (Fig. 15D) [203]. Amphiphilic triblock copolymer Pluronic F127 is a clinically approved soluble polymer that is readily soluble in both organic and aqueous solvents [204]. Kim et al. [205] prepared soluble MN by dissolving hydrophilic antigen and hydrophobic adjuvant Toll-like receptor 7/8 (TLR7/8) agonist (R848) in Pluronic F127 for cancer immunotherapy (Fig. 16). Nanomicelles (NMCs; 30 to 40 nm) could be formed after subcutaneous dissolution of MN to enhance the delivery of antigen and R848. In vivo experiments in mice have shown that MN administration can effectively enhance antigen-specific humoral and cellular immune responses, resulting in excellent antitumor effects. Using a layer-by-layer (LBL) method to prepare a rapidly detachable coating on the charge-reversible polymer PDM MN for delivery of the model vaccine antigen ovalbumin (OVA) can achieve efficient immune responses and is expected to be used in various immunotherapeutic strategies [206].

### Medical beauty

Solid MNs have been approved for utility as a cosmetic tool, and the induction of elastin and collagen expression and deposition through mechanical stimulation of MN has achieved skin rejuvenation effects and can also facilitate more efficient delivery of cosmetic ingredients such as HA into the skin to achieve the effect of moisturizing and smoothing wrinkles (Fig. 15E) [207]. Through clinical studies on HA-MN patches loaded with arginine/lysine peptides, acetyl octapeptide-3, palmitoyl tripeptide-5, adenosine, and seaweed extracts, it was found that HA-MN could significantly reduce the fine lines/wrinkles of subjects, improve the skin hydration, increase the density and thickness of dermal skin, promote skin elasticity/firmness, and have significant anti-aging effect [208]. Jang et al. [209] prepared adenosine-loaded high and low molecular weight HA patches (Ad-HMN and Ad-LMN, respectively) to explore their anti-aging effect. The 12-week clinical test results showed that both HA patches did not cause any adverse skin reactions, while the Ad-HMN group could effectively reduce skin wrinkles, enhance skin elasticity and density, and have a good anti-aging effect. It is believed that MNT with HA MN patches is a novel cosmetic strategy with valuable potential. Nowadays, the cosmetic and cosmeceutical industries are developing MN eye masks and MN skin care masks to relieve skin aging, blemishes, pimples, and various skin problems/diseases, which indicates the huge value and tremendous potential of the MN applicability.

## Properties

Generally, MNs should puncture the skin barrier to form channels ensuring penetration of target molecules and signals in a minimally invasive and painless style [210]. Unique properties of MNs include the semi-implantable ability, specific stimulus response mechanism, and capability of extracting both surface and internal information, in addition to antibacteria, sticking ability, swellability, dissolvability, etc. Nonetheless, capabilities of versatile operation on different organs, durable wearability, rapid transmission, sensitive extraction of skin ISF molecules, and signals still require improvement [211]. Furthermore, advanced novelties are awaiting development for carrying out comprehensive, intelligent, and multifunctional applications.

### Semi-implantable ability

MNs are semi-implantable with base on surface and needle tips under skin, which can realize monitoring and regulation of both body surface and internal environment, not only for drug delivery, electrophysiological recording, real-time monitoring, and diagnosis but also for regulation of intracellular activities [212]. Semi-implantable MNs can meet the growing technical demand for accurate detection or regulation of biological activities and also provide an ideal platform for external integration of complex functions and electronic integration [213]. Although less defined and summarized than the critically acclaimed noninvasive and fully implantable bioelectronics, semi-implantable MNs has emerged as a highly unique technology driving the development of biochips and smart wearable devices.

### Stimulation

MNs can stimulate tissue regeneration, enhance healing ability, and stimulate skin metabolism [214]. Physical stimulation of MNs can activate the damage repair mechanism, stimulate the release of fibroblast growth factor (FGF), platelet growth factor (PGF), and transforming growth factor α and β (TGF-α and TGF-β, respectively), up-regulate the expression of hyaluronic synthase, activate cytokines with growth factors, and stimulate the release of FGF, PGF, TGF-α, and TGF-β [215]. MN stimulation can up-regulate the expression of type I and III collagen, promote the proliferation of collagen and elastic fibers, release the hardened collagen fibers, and reconstruct scar/damage tissue, so as to achieve therapeutic, skin regenerative and anti-aging effect [216]. Besides, physical signals/photosignals/electrosignals and drug dose on MNs can perform targeted stimulation pattern correspondingly [217].

### Response performance

The MNs can respond to signals including light, electric, pH variation, physiological parameter changes, and recommended dose level when loading functional ingredients [218]. For example, PDT and PTT using MNs loaded with photosensitive and photothermal agents show good response to NIR light-inducing photodynamic and photothermal treatment, respectively [219]. Besides, NIR-triggered TDD is controllable to accommodate heat-induced melting of micro/nano-capsule shell to release drug/ingredient core [220]. Specific release of drugs or other ingredients upon response to certain signals can be controlled to prevent side effects from excessive release or unsatisfactory release [221]. Polymers sensitive to pH (e.g., swell/dissolve under certain pH change) can be used in pH-responsive releasing drugs/ingredients during specific pH variation [222]. Electrochemical control of pH is a tool to trigger such release. Intelligent response of certain level of molecules (e.g., glucose, cytokine, histamine, and ROS) requires specific recognition reaction as a switch of drug release [223].

### Antibacteria and anti-inflammation

Antibacterial MNs mainly use antibacterial materials such as chitosan, silver NPs, antibacterial peptides, positive ammonium salts, antibacterial herbs, bioactive extracts, and other antibacterial ingredients [224]. Anti-inflammation MN can perform the elimination of the inflammatory factors generated from hyperimmune reaction, reducing the painful inflammatory response [225]. Antibacteria effect is often combined with anti-inflammatory effect as the wound dressing/bandage to promote wound healing. For chronic nonhealing wounds such as diabetic foot, better curative effect can be achieved by using MN for multi-effect synergistic treatment such as antibacterial, anti-inflammatory, and promoting angiogenesis [226,227].

### Swellability/dissolvability

Swellable MNs can interact with skin ISF and swell to form strong interlocking with tissue [228]. They mostly consist of swellable polymers or hydrogels. With volume expansion caused by liquid adsorption, the MNs can obtain strong mechanical interlocking and flexible adhesion with tissue [229]. The tissue adhesion strengths of swellable MNs are much higher than nonswellable MNs. Dissolvable MNs can rapidly release drug/ingredients during the dissolving process of MN tips [230]. Drugs in MN tips can be totally released, preventing incomplete release, which causes drug waste. Moreover, dissolvable MNs do not leave fractured MN tips or remain inside skin, reducing the risk of needle breakage damage. Furthermore, infection risk can be notably lowered since the penetrated epidermis can recover to normal skin state without MNs. These MN tips usually employ dissolvable materials such as PVA, HA, PVP, carboxymethyl cellulose (CMC), PGA, and alginate, which are biocompatible, water-soluble, nontoxic, noncorrosive, nonirritating, or safe from natural organisms [231].

### Sticking ability, conductivity, and others

In addition to swelling adhesion, sticking materials or special structures can also lead to sticking ability [232]. Adhesive hydrogels such as chitosan, gelatin, and PDA gels are appropriate choices for MN supporting layers [233]. In terms of specific structures, bionic microsuction cups and geometric protrusions are successfully adopted to promote the sticking ability of MNs [234,235]. When applied to skin, the MN tips are inserted into epidermis, with the base plane left on surface. The base plane plays a useful role as the drug reservoir when it needs to retain sufficient drug supply during the TDD process. On the other hand, the supportive base plane of separable MNs can remove from MN tips to shorten the wear time and prevent the possible inconvenience [236]. Notably, conductive, luminescent, and catalytic properties would be introduced into MNs to conduct photoelectronic therapy, combining multifunctional treatment such as electrical stimulation, electroluminescent, PTT, PDT, and catalytic therapies [237–239].

## Prospective Outlooks and Challenges

As a rising star, MNs are generating novel strategies for multifunctional treatment, diagnosis, monitors, beauty, and optoelectronic devices, combining multidisciplinary basis upon biomedical, nanomedical, photonic, electronic, photoelectronic, catalytic, and nanomaterial principles, for the sake of improving human health and quality of living. With the aid of multifunctional MNT, various diseases such as cancer, diabetes, cardiac–cerebral vascular disease, wound infection, and contagions could have diagnosis and therapy routes. In a similar fashion, human immunodeficiency disease, coronavirus disease 2019 (COVID-19), and other difficult diseases will have high possibility of immunization, diagnosis, and therapy. However, the industrialization of pharmaceutical products requires a long process. Several improvements may speed up this process.

### Feasibility

Approved drugs and materials have been clinically and lawfully permitted for in vivo use, which are more secure in terms of biosafety, and thus, MNs prepared using these substances are more likely to be approved for applications in clinical trials, contributing to the early realization of MNT.

To shorten the time of complex pharmacological research, PDT and PTT based on photosensitized and photothermal materials/NPs are rising fields adopting biosafe component/nanostructures that catalytically engender ROS and energy, respectively, to kill viruses, microbials, pathogens, and malignant cells.

Personalized and customized MNs are designed to accommodate that MNs can be prepared at home, beauty salon, and health consulting and clinic centers. The prepolymer solutions containing different active ingredients can be purchased directly, and people only need to add the ingredient solution to a small MN preparation machine to prepare MN patches loaded with active ingredients for the treatment of corresponding diseases, healthcare, and beauty and skin problems.

### Flexibility

The design of flexible MNs that can support electronic/optoelectronic devices will become a future trend. The existing MN patches are generally small in size and can be directly pressed into the skin for real-time monitoring, diagnosis, information transmission, human–machine interaction, and controllable treatment. Designing an MN patch with a larger size can not only effectively increase the content of active ingredients but also facilitate the functional integration of the MN patch with larger area. It is also necessary to develop the insertion device optimizing the MN patch to achieve optimal attachment. For example, the flexible MN batteries using biocompatible electrolyte (e.g., ISF and serum) and electrodes (e.g., Ti alloy, Co alloy, noble metals) could be developed to power the implantable and wearable devices such as heart pacemakers, body chips, artificial electronic cochlear implant, wearable sensors, and electronic tablets, avoiding the hazard risk of nonbiocompatible corrosive and poisonous electrolyte from Li-ion batteries otherwise. The unique semi-implantable property enables the MN sensors to monitor both surface and internal bioinformation simultaneously.

### Intelligentization

Intelligent MN patches with highly integrated functions are awaiting development. The combination of MN patch and intelligent electronics can not only realize real-time health monitoring, remote control, and big-data healthcare but also take corresponding disease treatment or countermeasures against abnormal conditions. For example, the MN patch can monitor the blood glucose value in the body in real time and automatically deliver an appropriate amount of insulin to the body when the blood glucose value exceeds a certain threshold to reduce blood glucose. Through real-time monitoring of the electrocardiogram of patients with angina pectoris, if abnormal heart rate occurs, the MN patch can deliver the loaded angina pectoris emergency medicine (e.g., nitroglycerin) to the body intelligently, which can greatly reduce the mortality of patients. It is believed that the intelligent MN patches will greatly benefit the healthcare and quality of human life.

Flourishing development of brain–computer interfaces, artificial neural network, implantable/wearable devices, artificial intelligence, and software robotics requires the intimate alliance, interaction, and integration between technology and living organisms. Because the basic building blocks of living organisms are different from those used in electronic devices, the ability to connect artificial devices to biological systems is critical to the success in these fields. MN chips could be expected to have the integrated structure of perception, storage and computing, large-scale parallel processing, and event-driven operation, enabling the accurate perception, response, and treatment to real-time events in a robust and energy-efficient manner. MNs as the integrated platform for efficient and low-power processing of big data could combine neuromorphic system to build a biomimetic cognitive system, matching the biological neural perception and processing capabilities. MN chips combining the biological/artificial neural networks could perform tasks such as pattern classification and feature extraction to analyze the photosignals/electrosignals/chemical signals of bioinformation with real-time solving problems of disease, accidents, uncertain conditions, and abnormal states.

Programmable photoelectronic MNs combine the polarized light, infrared treatment, and stereo-dynamic interference electrical therapy, which are highly integrated, and can be carried out independently or at the same time, almost targeted at all pain lesions. They show advantages including no pain, no injury, no risk of infection, short treatment time, wide range of adaptation, no side effects, and complications. Polarized light emitted by MNs can be used in elderly patients who have allergic reactions to drugs and hemorrhagic symptom and other patients who are not suitable for nerve block therapy. Infrared polarized light emitted by MNs can be used as an alternative therapy for nerve block to treat exercise pain (a variety of subacute chronic muscle and joint pain), pain caused by various bone and joint degenerative diseases (e.g., cervical spondylosis and periarthritis of shoulder), and various arthritis. Additionally, infrared MNs can reduce inflammation and improve the immune ability and disease resistance. Combination of various wavelength lasers can perform better than single-wavelength laser for therapy. Besides, stereo-dynamic interference electro-therapeutic MNs can relieve pain and quickly eliminate depression pain or exercise fatigue. Hence, the programmable photoelectronic MNs combining polarized light, infrared, and stereo-dynamic interference electrical therapy can process the logic encoding of diverse photoelectric therapeutic pathways to adapt to different conditions and optimize the therapy efficacy.

### Integrated systems

MN chips refer to integrated platform that is implantable/wearable to body and capable of collecting photo-/electro-/chemical signals, analyzing, computing, and processing treatment in real time. According to diverse functions, the MN chips could be classified into DNA chips, RNA chips, protein chips, tissue chips, organ chips, neural chips, multifunctional chips, etc. The MN chips could load high density of precisely fixed biomolecules, integrated circuits, and devices, which show advantages of high throughput, miniaturization, and fast processing speed, and accordingly could be widely used in the fields of cell analysis, disease diagnosis, drug screening, individualized therapy, and gene sequencing. For example, the MN RNA chips could perform RNA sequencing and detection for the real-time virus (e.g., COVID-19) detection, analysis, diagnosis, treatment, drug screening, and delivery to ensure the security of personal health safety. The MN cell chips using breast cancer-specific peptide as probe molecules could perform breast cancer cell detection in real time. MN organ chips could realize the dynamic monitoring of normal/abnormal signals of certain organs and the drug metabolism/efficacy pathways on target organs. The development of MN chips depends largely on the multidisciplinary integration of multiple technologies. The pursuit of higher detection sensitivity, lower detection cost, and more refined detection/treatment performance is the unlimited driving force for the upgrading of MN chip technology. The wearable/implantable MN chips could be promising for real-time monitoring/diagnosis/treatment of either body surface or internal organs. The advantages of implantable MN chips include the remote sensing and control of biological information, which can be implanted into human body and perform a series of tasks. The future of MN chips requires the combination of novel techniques such as biological computers and artificial intelligence to achieve biological information extraction, data storage, computing, processing, intelligent decision-making, and instruction execution.

### Optogenetics

Optogenetic MNs could possess advantages of noninvasive, reversible, and spatial–temporal precision, offering novel approach and concepts for disease treatment. As a good inducer of gene expression, light can manipulate gene expression and cell behavior with unprecedented spatial and temporal precision. With further exploration of optogenetic MN techniques, the personalized precision therapy and clinical transformation based on optogenetics could become possible. Programmable light-emitting MNs as the optogenetic tools can correspond to different light wavelengths for applications in precision therapy for the neurological diseases, tumors, cardiovascular diseases, diabetes, and intestinal diseases, as well as applications for controlling gene transcription expression, gene editing, gene recombination, and organelle movement. At the same time, multifunctional combination of optogenetic MNs and intelligent electronic equipment will be used in implantable/portable intelligent bioelectronic medicine, artificial intelligence diagnosis, and treatment, which are promising to be realized. The remote controllability, reversibility, and nontoxicity of the optical control system provide a solid foundation for the application of optogenetics in biomedicine. The success of these approaches will have a long-lasting influence on precision medicine for practice in future.

### Bioelectrodes, energy, and others

MN electrodes applied with certain electric potentials could induce electrochemical catalytic reactions to reduce inflammatory factors, remove vascular thrombosis, and eliminate gastric alcohol or other target substances in organs, in vitro and in vivo. Subsequently, MN bioelectrodes could treat a variety of diseases through electrical stimulation therapy and bioelectrocatalytic reactions, such as electrocatalytic oxygen evolution reaction for blood oxygen supply and hydrogen evolution reaction to reduce peroxide inflammatory cytokines avoiding the tissue damage or aging. Besides, MN biofuel cells or biobatteries with biocompatible electrolytes are demanded for wearable and implantable devices (heart pacemakers, body chips, electronic cochlear, electronic tablets, etc.), avoiding the poisonous and corrosive electrolytes from Li-ion batteries, and especially avoiding the tedious surgery every few years to replace battery of heart pacemakers. Bioenergy from inner or surface system could be directly converted into electric power by MN biofuel cells. Safe and secure electrodes could be guaranteed to eliminate hazard risks. Various other applications such as the microplastic beauty, microdisplay light-emitting devices, and programmable matrix array devices are awaiting for future exploration to benefit scientific research and industrial development.

## Concluding Remarks

Owing to the rapid development of micromachining technology, growing studies of MNs are creating approaches for multifunctional therapy, diagnosis, monitoring, immunization, medical beauty, electronic/optoelectronic devices, intelligent integrated circuits, and so on. Combining the advantages of multidisciplinary techniques including biomedical, nanomedical, photonic, electronic, photoelectronic, catalytic, and nanomaterial technologies, optogenetics, electronic information, computing, and artificial intelligence, MNs become promising to improve human health and quality of life, expanding to various intelligent devices and facilities. Accordingly, numerous diseases such as cancer, diabetes, cardiac–cerebral vascular disease, wound infection, contagions, and arthritis could be healed by the multifunctional MN diagnosis and therapy. Ascribed to the various merits including painless, minimally invasive, flexible, portable, simple operation, precise control, accurate detection, and strong adaptability, MNs will become a significant future trend among hotspots involving nanotechnology, flexible devices, photo-/electro-chemical catalytic therapy, optogenetics, remote control, healthcare, big data, human–machine interaction, brain–computer interfaces, artificial neural networks, Internet plus artificial intelligence, and so on.

Programmable, intelligent, and photoelectronic MNs allying PDT, PTT, polarized infrared/photoelectronic devices, integrated circuits, and nanotechnology could process the logic encoding of diverse monitoring and treatment pathways to adapt to different conditions, optimize the therapy efficacy, realize the real-time health monitoring, remote control, and big-data healthcare, and also take instant disease treatment. MN chips show advantages of high throughput, miniaturization, and fast processing speed and could be used in cell analysis, disease diagnosis, drug screening, individualized therapy, nucleic acid detection, and RNA/DNA sequencing. MN chips combining the biological/artificial neural networks and neuromorphic system for efficient and low-power processing of big data could build a biomimetic cognitive system, matching the biological neural perception and processing capabilities. Programmable photoelectronic MNs as the intelligent optogenetic tools can control different light wavelengths for precision therapy of neurological diseases, tumors, cardiovascular disease, diabetes, and intestinal diseases, as well as applications for the control of gene transcription expression, gene editing, gene recombination, and organelle movement.
